# Myelin sheaths persist for weeks following axon degeneration

**DOI:** 10.1126/sciadv.aeb2628

**Published:** 2026-03-04

**Authors:** Megan E. Doty, Edward C. Tuckerman, Enrique T. Piedra, Robert A. Hill

**Affiliations:** Department of Biological Sciences, Dartmouth College, Hanover, NH, USA.

## Abstract

Axon degeneration underlies clinical deficits in traumatic injuries and neurodegenerative disease. It is not clear how myelinating oligodendrocytes are directly affected by or respond to axon injury and loss. Here, we combined intravital imaging with laser axotomy or single neuron ablation to determine the longitudinal responses by oligodendrocytes that myelinate the degenerating axon. We find that while axons rapidly degenerate, myelin sheaths devoid of axon can persist for weeks. These remaining myelin sheaths lose compaction and slowly shrink. Local to the injury, oligodendrocyte homeostasis is largely maintained, with only a brief change in myelin sheath structural plasticity. After neuron ablation and axotomy, clearance of axon debris is delayed if the axon is myelinated. However, longitudinal imaging of microglia revealed only rare microglial engagement with injured axons, regardless of myelination status. Likewise, microglia did not engage with de-axoned myelin sheaths. These findings highlight the stability of myelinating oligodendrocytes and provide insight into homeostatic neuroglia responses following injury.

## Teaser

Axon degeneration results in persistent, empty myelin sheaths and elicits unexpectedly rare phagocytic responses from microglia.

## INTRODUCTION

Clinical deficits following traumatic brain injury (TBI) or in neurodegeneration are causatively linked to axon damage and loss (*1*–*8*). In TBI, mechanical stress can directly transect axons or disrupt cytoskeletal organization to induce a degenerative cascade (*2*, *9*). In aging and neurodegenerative disease, a plethora of molecular and cellular changes induce axonal stress, damage, and subsequent degeneration (*7*). Following an insult, axon degeneration proceeds through a highly conserved mechanism known as Wallerian degeneration (*8*, *10*). This can lead to the loss of long-range projecting axons, resulting in broad network dysfunction affecting cognitive or motor function (*11*–*15*). Likewise, loss of locally projecting axons of parvalbumin-expressing (PV+) interneurons causes neural circuit disruption (*16*–*22*).

Myelinated axon populations in the mammalian cerebral cortex are a mix of long-range and local glutamatergic and PV+ axons (*23*, *24*). Myelin is a compact multilayer lipid membrane that surrounds axons to improve action potential efficiency and provide metabolic support. In the peripheral nervous system (PNS), a myelinating Schwann cell ensheathes a single axon. Schwann cells actively contribute to the clearance of their axon after injury before being cleared themselves (*25*–*29*). In contrast, in the central nervous system (CNS), myelinating oligodendrocytes can ensheathe up to 60 axons (*30*, *31*). How oligodendrocytes respond to the injury of an axon and how this might affect myelin homeostasis locally is not well understood. In the CNS, myelin pathology is reported on surviving axons following TBI and in neurodegeneration, including abnormal myelin outfoldings and demyelination (*32*–*38*). Variable oligodendrocyte responses, including death (*39*, *35*), quiescence (*39*, *40*), or normal homeostasis (*34*, *40*), have been reported following axon degeneration induced with enucleation, optic nerve crush, or TBI. In addition, myelin sheaths lacking immunoreactivity for, or ultrastructural evidence of, an axon have been described in degenerating human and aging monkey tissues (*41*–*43*). These findings of myelin pathology originate from broad injuries, making it difficult to determine if myelin pathology results from axon injury directly. With variability in these data and a lack of longitudinal information, the oligodendrocyte response to axon degeneration remains an open and intriguing question.

To investigate the longitudinal cellular dynamics of axonal degeneration and/or regeneration of neocortical axons, previous work has used laser axotomy combined with intravital optical imaging (*44*, *45*). The myelination status of these axons and the response by surrounding myelinating oligodendrocytes were not investigated. Moreover, it is known that microglial cells exhibit a transient response to the axonal injury site without other evidence of glial scar formation (*45*–*47*). However, little else is known about glial responses to damaged and degenerating axons and how the cellular debris is cleared.

Here, using laser axotomy or single neuron ablation combined with intravital imaging, we discover a remarkable stability of myelin sheaths and surrounding oligodendrocytes following axon degeneration. Myelin sheaths persist long after the loss of their axon, with a loss of compaction and a gradual loss of length. This has little to no effect on the homeostasis of local myelin sheaths and oligodendrocytes. In addition, we find little evidence of microglial response to or engagement with injured and degenerating axons. Despite myelin delaying clearance of axon debris, myelination status was unimportant for microglia engagement during degeneration. Furthermore, we find no evidence of microglia engagement with myelin sheaths devoid of axons. Together, these data demonstrate an unexpected stability of the cortical environment and precise homeostatic response by myelinating oligodendrocytes following the injury and loss of single axons.

## RESULTS

### Myelinated axons retract and degenerate following axotomy

**Fig. 1.**
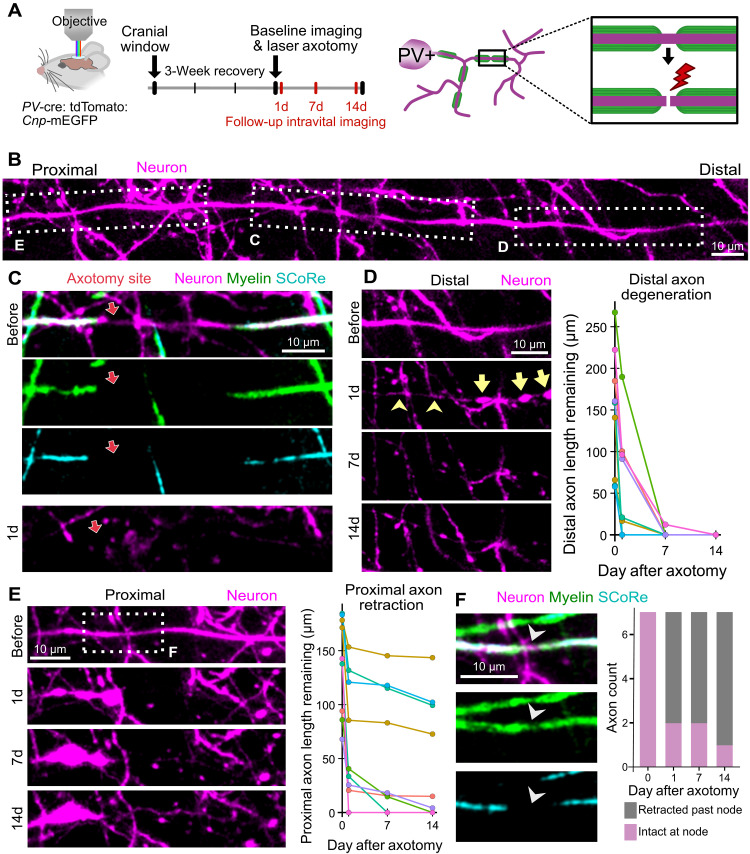
Myelinated PV+ axons degenerate rapidly following axotomy. (**A**) Schematic and timeline for laser axotomy and longitudinal intravital imaging. (**B**) Representative intravital image of an axon targeted for axotomy, acquired before laser axotomy. (**C**) Intravital image showing the axotomy site (red arrow) before and 1 day after the axotomy. Axotomy was targeted to a break in myelin (28.6 μm), as shown with membrane-tethered EGFP expressed in oligodendrocytes (*Cnp*-mEGFP) and SCoRe microscopy. (**D**) Image series shows retraction and later degeneration of the axon distal to the axotomy site. Yellow arrowheads point to a thin segment of axon remaining, and yellow arrows point to axon spheroids, connected by thinner axonal segments. On the right, axon degeneration is quantified as the length of axon remaining within the imaging field of view, the color of each line corresponds to the animal. (**E**) Image series showing retraction of the axon proximal to the axotomy site. Graph represents the length of axon remaining within the imaging field of view, and the color of each line corresponds to the animal. (**F**) Boxed region from (E) showing the node of Ranvier (white arrowhead) proximal to the axotomy site, before the axotomy. The number of proximal axon stumps that retract past their proximal node of Ranvier is shown on the right (*n* = 7 axotomies with proximal nodes in the image volume from six mice). For (D) and (E), *n* = 9 axotomies from seven mice. d, day.

To determine how myelinating oligodendrocytes respond to axon loss, we performed longitudinal intravital imaging of layer I of the somatosensory cortex through a cranial window and transected axons with a laser-mediated axotomy. To visualize myelin and axons, we used a triple-transgenic mouse line that expresses tdTomato in PV+ interneurons (*Pv*-cre: tdTomato) and membrane-anchored enhanced green fluorescent protein in mature oligodendrocytes (*Cnp*-mEGFP). PV+ interneurons are an abundant population of myelinated axons in the cortex, and their activity is key for neuronal circuit function (*23*, *48*). With *Cnp*-mEGFP and spectral confocal reflectance (SCoRe) microscopy (*49*), for label-free visualization of compact myelin, we identified nodes of Ranvier or other breaks in myelin as target sites for axotomies (Fig. 1, A to C). By targeting nodes (1- to 2-μm-wide break in *Cnp*-mEGFP signal) or breaks in myelin (22- to 46-μm-wide break in *Cnp*-mEGFP signal), we aimed to minimize direct damage to the surrounding myelin. While laser-induced axotomies are commonly used for single axon injuries, nonspecific damage to nearby tissue inevitably occurs as well. We minimized the duration of laser exposure necessary to induce a transection to limit this nonspecific damage.

We first characterized degeneration of myelinated PV+ axons following axotomy. Previous axotomy studies of the mammalian CNS describe axon degeneration both proximal and distal to the axotomy site (*44*, *45*, *50*–*54*). These studies focused on presumed unmyelinated axons of the cerebral cortex (*44*, *45*) or were not selective for unmyelinated injury sites in the spinal cord (*50*–*54*). Although we cannot know for certain which region of the axon is proximal and which is distal relative to the axotomy site, axon stumps can be classified as distal or proximal based on their pattern and timing of degeneration. While proximal and distal axon stumps both retract acutely after axotomy, the remaining distal axon later undergoes Wallerian degeneration (*50*). Consistent with this, we observed axon retraction distal to the injury site in the first day after axotomy, followed by complete loss of the distal axon segment within the image field of view (Fig. 1D; *n* = 9 axons from seven mice). Proximal to the axotomy site, we also observed axon retraction in the first day after injury and only minor additional retraction in the 2 weeks that followed (Fig. 1E; *n* = 9 axons from seven mice). Of the nine axons targeted for axotomy, seven had nodes of Ranvier discernible within the image field of view proximal to the axotomy site. Proximal axon stumps often retracted past their proximal node (Fig. 1F and fig. S1). Therefore, the distance retracted by the proximal axon stump often exceeds the length of the myelin sheath adjacent to the injury site. Together, transected myelinated axons rapidly degenerate, resulting in a loss of axons in territories occupied by their myelin sheaths.

### De-axoned myelin sheaths persist for weeks

**Fig. 2.**
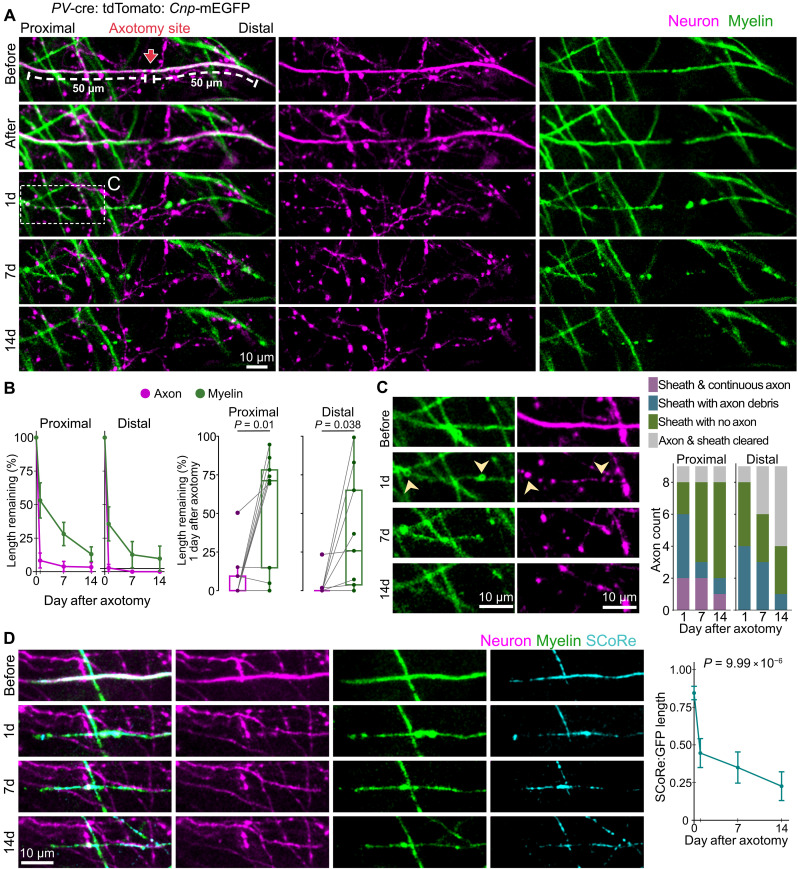
Myelin sheaths persist after axon loss. (**A**) Image series of rapid axon loss and gradual myelin loss after axotomy. ROIs (50 μm) used for quantification are indicated on either side of the axotomy site. (**B**) Axon and myelin changes after axotomy, represented as a percentage of baseline length remaining. Line graphs show loss throughout 2 weeks after axotomy [mean ± SEM, from two-way repeated measures analysis of variance (ANOVA), interaction term *P* = 0.008 for proximal ROI and interaction term *P* = 0.04 for distal ROI]. Box plots show paired axons and myelin remaining 1 day after axotomy (significance determined from paired *t* test). (**C**) Boxed region from (A) showing a de-axoned myelin sheath with axon debris (1 day, yellow arrowheads) and without axon debris (7 days). For the entire lesion-adjacent myelin sheath, the frequency of persistent de-axoned myelin is represented on the right. (**D**) Image series of a de-axoned myelin sheath that remains 14 days after axotomy, with SCoRe as an indicator of myelin compaction. The sheath shown here is distal to the axotomy site shown in Fig. 1C. Line graph shows SCoRe: GFP after axon loss (mean ± SEM, *n* = 7 myelin sheaths from five mice, significance determined from repeated measures ANOVA). For (B) and (C), *n* = 9 axotomies from seven mice. Detailed statistical results in data S1.

We next investigated changes to myelin sheaths in response to axon loss or injury. To do so, we compared the degeneration patterns of axons to their myelin sheaths by measuring the length of each structure in 50-μm regions of interest (ROIs) on either side of the axotomy site (Fig. 2A). Fifty microns is shorter than the length of each myelin sheath analyzed; therefore, this ROI is specific to the individual myelin sheath adjacent to the axotomy. We found that myelin sheaths are lost at a slower rate than axons after axotomy, both proximal and distal to the axotomy site (Fig. 2, A and B, and fig. S1). This difference is most pronounced 1 day after axotomy, when most axons have retracted more than 50 μm (six of nine proximal and seven of nine distal axons), yet most myelin sheaths remain (eight of nine proximal and seven of nine distal myelin sheaths) (Fig. 2B and fig. S1F). Unexpectedly, even 14 days after axotomy, persistent myelin was observed both proximal and distal to the axotomy site. We termed this persistent myelin devoid of an axon as de-axoned myelin.

After axon retraction or degeneration, we noted discontinuous axon debris or fragments associated with persistent myelin sheaths (Fig. 2C and fig. S1C). To look at the prevalence of this phenomenon and how it changes over time, we classified the entire myelin sheath adjacent to the axotomy site as sheath with continuous axon, sheath with axon debris, sheath with no axon, or axon and sheath both cleared (Fig. 2C). Myelin sheaths with continuous axons were only observed proximal to the axotomy site (Fig. 2C). Myelin sheaths with axon debris were observed both proximal and distal to the axotomy site, and the frequency of this observation decreased over time. Consistent with this, on the proximal side, observations of myelin sheaths without axon debris increased over time, concordant with the clearance or dissolution of axon debris (Fig. 2C). On the distal side, observations of myelin sheaths without debris decreased during the first week and did not change during the second, along with increased observations of cleared myelin sheaths (Fig. 2C).

Next, we measured SCoRe length as a ratio to mEGFP length to assess myelin sheath compaction (Fig. 2D). Here, we focused on the seven myelin sheaths that remained in the 50-μm ROIs 14 days after axotomy (five of nine proximal, two of nine distal). We observed a significant loss of SCoRe in de-axoned myelin sheaths, with the most pronounced decrease occurring the first day after axotomy (Fig. 2D and fig. S1). This indicates myelin decompaction after axon loss.

The image volume for four of nine transected axons included a second distal internode, allowing us to characterize de-axoned myelin sheaths one internode away from the axotomy site (fig. S2A). Like lesion-adjacent internodes, the myelin sheath persisted after axon loss, with a gradual loss of length and loss of compaction (fig. S2B). In addition, image volumes for five of nine transected axons included a second proximal internode (fig. S2C). Axon retraction beyond the second proximal internode was minor, allowing us to characterize myelin on remaining stumps of injured axons (fig. S2C). In the territory occupied by the second proximal internode, myelin loss was comparable to axon loss (fig. S2C). In addition, myelin compaction, as indicated by SCoRe, remained mostly normal, with no significant decrease during the 2 weeks after axotomy (fig. S2C). Together, these data demonstrate the stability of myelin after axon injury, with notable persistence of de-axoned myelin sheaths that progressively lose their integrity.

### Oligodendrocytes maintain homeostasis after axotomy

**Fig. 3.**
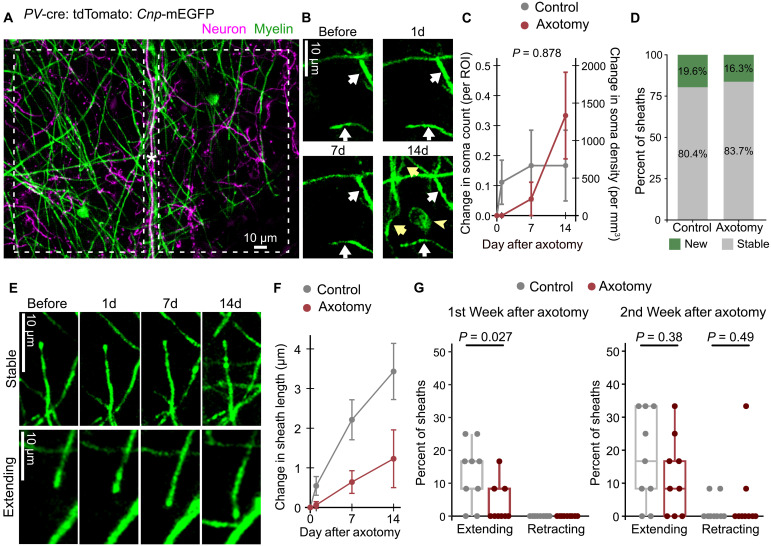
Oligodendrocytes largely maintain homeostasis local to axotomy. (**A**) Dotted line boxes indicate ROIs used to analyze oligodendrocyte homeostasis in an axotomy position. Asterisk indicates the axotomy site. (**B**) Image series shows the generation of a new oligodendrocyte (yellow arrowhead). Yellow arrows indicate new myelin sheaths, and white arrows indicate stable myelin sheaths that are present at baseline. (**C**) Quantification of the change in oligodendrocyte density (mean ± SEM, significance tested with two-way repeated measures ANOVA, shown *P* value compared control and axotomy positions). ROIs are 100 μm by 150 μm by 16.5 μm. (**D**) Distribution of new and stable sheaths in axotomy and control positions (data from 510 sheaths total). (**E**) Top image series shows a stable myelin sheath that does not change length. Bottom image series shows an extending myelin sheath. (**F**) Myelin sheath structural plasticity quantified as sheath length changes (mean ± SEM, tested with two-way repeated measures ANOVA, interaction term *P* = 0.0006). (**G**) Myelin sheath structural plasticity quantified as percent of myelin sheaths that are extending or retracting in each position, split into first and second week after axotomy (significance tested with Welch’s *t* test). For (C), (D), (F), and (G), *n* = 9 control and 9 axotomy positions from seven mice. Detailed statistical results in data S1.

Oligodendrocytes and myelin sheaths are generated from early development into late adulthood (*55*, *56*). To determine whether an axotomy affects homeostasis of oligodendrocytes locally, we analyzed oligodendrocyte generation and myelin sheath generation in the regions surrounding axotomized axons (Fig. 3A). Mice were 2 to 3 months of age at the time of axotomy, an age at which myelin generation and growth are observed in the cortex (*55*). Consistent with this, we observed the generation of new oligodendrocytes during the 2 weeks following axotomy (Fig. 3B). When compared to uninjured control positions, a local axotomy did not affect oligodendrocyte generation (Fig. 3C; *n* = 9 axotomy and 9 control positions from seven mice). Similarly, from random samples of myelin sheaths present at the end of the experiment, axotomy did not affect the prevalence of newly generated myelin sheaths (Fig. 3D; *n* = 510 sheaths from seven mice).

Oligodendrocyte homeostasis can also be reflected in myelin sheath plasticity. Existing myelin sheaths are typically stable, exhibiting no change in length; however, myelin sheaths can also dynamically extend or retract (Fig. 3E) (*55*, *56*). To determine whether a local axotomy affects myelin sheath plasticity, we measured changes in length for myelin sheaths that were present before the axotomy. A small positive change in myelin sheath length was observed in both axotomized and control positions, indicative of some myelin sheath extension (Fig. 3F). The average myelin sheath change in length was lower in axotomy positions in a time-dependent manner, compared to controls (Fig. 3F; *n* = 9 axotomy and 9 control positions from seven mice). Within each control and axotomy position, we classified myelin sheaths as extending, retracting, or stable and calculated the percentage of sheaths that exhibited each characteristic. In the first week after axotomy, we observed significantly fewer extending myelin sheaths in axotomized positions compared to uninjured control positions (Fig. 3G and fig. S3; *n* = 9 axotomy and 9 control positions from seven mice). In contrast, in the second week after axotomy, the distribution of extending, retracting, and stable myelin sheaths was comparable between axotomy and control positions (Fig. 3G and fig. S3; *n* = 9 axotomy and 9 control positions from seven mice). Together, oligodendrocyte homeostasis is largely unaffected by a local axotomy of a myelinated axon, with only a brief pause in myelin sheath extension.

### De-axoned myelin persists and oligodendrocytes maintain homeostasis after neuronal death

**Fig. 4.**
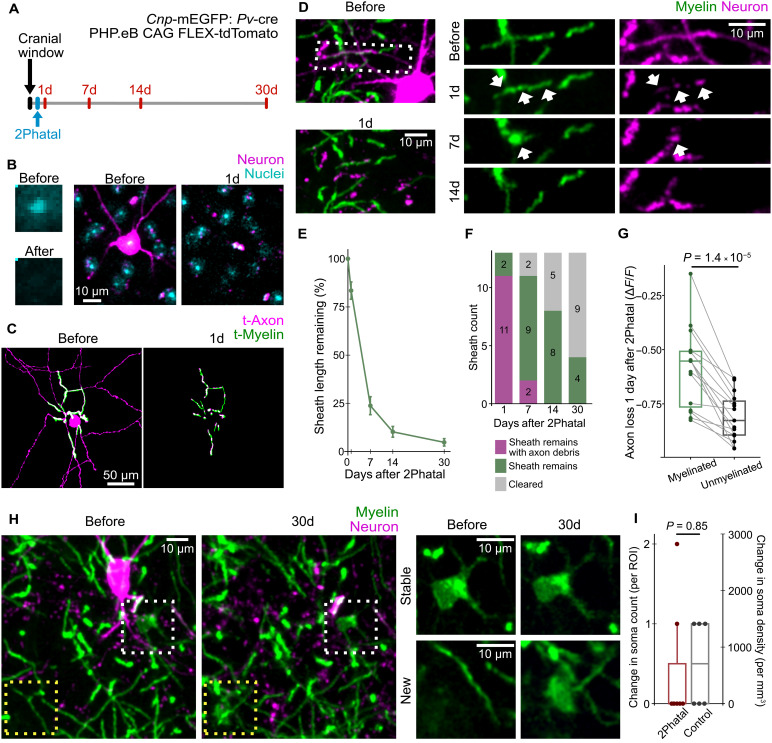
Myelin sheaths persist and delay axon debris clearance after neuron death. (**A**) Experimental timeline for neuronal ablation via 2Phatal and follow-up imaging for axon and myelin dynamics. (**B**) Before and after images show photobleaching of nuclear dye during 2Phatal. Images on the right show clearance of a targeted neuron within 1 day of 2Phatal. (**C**) Example trace of soma, axon, and myelin sheaths of a neuron targeted for 2Phatal. (**D**) Image series of a neuron targeted for 2Phatal, including its axon and an associated myelin sheath. White arrows indicate axon debris under the myelin sheath that remains after neuron death. (**E**) Myelin sheath length remaining is quantified as a percentage of baseline (mean ± SEM, *n* = 13 myelin sheaths from five neurons in four mice). (**F**) Frequency of persistent myelin with and without axon debris. (**G**) Paired comparisons of axon clearance in adjacent myelinated and unmyelinated axon segments (*n* = 15 axon segment pairs from six neurons in five mice, significance tested with paired *t* test). (**H**) Image series shows oligodendrocytes and myelin near a targeted neuron before and 30 days after 2Phatal. White box indicates a stable oligodendrocyte. Yellow box indicates the formation of a new oligodendrocyte. (**I**) Quantified change in oligodendrocyte density 30 days after 2Phatal relative to baseline (*n* = 7 2Phatal and 6 control positions from four mice, significance tested with Welch’s *t* test). ROIs are 150 μm by 150 μm by 31.5 μm. Detailed statistical results in data S1.

We next determined whether de-axoned myelin sheaths persist after axons are lost following neuron death. We induced apoptosis using 2Phatal, whereby photobleaching of Hoechst nuclear dye leads to apoptosis and clearance of neurons within 1 day (Fig. 4, A and B) (*57*). In contrast to laser-mediated axotomy or laser-mediated thermal ablation, 2Phatal does not induce cell rupture, spillage of cytosolic materials, or damage to surrounding structures (*57*). We used a dual transgenic mouse line (*Cnp*-mEGFP: *Pv*-cre) and low-titer intravenous viral delivery (PHP.eB-Flex-tdTomato) to achieve sparse fluorescent labeling of PV+ interneurons. This labeling strategy provided a clear visualization of neuronal processes, allowing for the confident tracing of the axon of target layer II/III PV+ interneurons to identify associated myelin sheaths (Fig. 4C and fig. S4, A to C). All analyzed myelin sheaths were approximately parallel to the pial surface to provide confidence in measurements. Notably, 1 day after 2Phatal, all myelin sheaths associated with cleared neurons remained (Fig. 4, C to F, and fig. S4; *n* = 13 myelin sheaths from five neurons from four mice). These de-axoned myelin sheaths gradually lost length in the weeks following 2Phatal, consistent with observations after axotomy (Fig. 4, D and E, and fig. S4B). While most myelin sheaths were cleared within 30 days after 2Phatal, small regions of myelin remained for 4 of 13 sheaths (Fig. 4, E and F, and fig. S4B).

Apoptotic neurons were cleared within 1 day of 2Phatal; however, we noticed small fragments of axon debris lingering within myelin sheaths (Fig. 4D). This lingering debris was not observed in territories previously occupied by unmyelinated regions of axons or dendrites. To quantify this, we compared the change in tdTomato fluorescence in directly adjacent myelinated and unmyelinated regions of an axon, 1 day after 2Phatal. We found significantly greater loss of tdTomato fluorescence in unmyelinated regions of axons (Fig. 4G and fig. S4D; *n* = 15 myelinated and paired unmyelinated axon segments from six neurons in five mice). These data confirm the persistence of myelin sheaths after axon loss and suggest that myelin sheaths may delay clearance of axon debris during degeneration.

We also analyzed the density of oligodendrocyte somas in regions surrounding targeted neurons compared to control positions. All oligodendrocyte somas present before 2Phatal maintained a healthy appearance at 30 days after neuron death (Fig. 4H). In addition, the generation of new oligodendrocytes in regions surrounding targeted neurons was comparable to what was observed in control positions (Fig. 4, H and I, and fig. S4D; *n* = 7 2Phatal and 6 control positions from four mice). These data indicate that the death of a single myelinated neuron does not affect the local homeostasis of oligodendrocytes.

### Microglia acutely respond to axotomy sites but rarely engage with injured axons

**Fig. 5.**
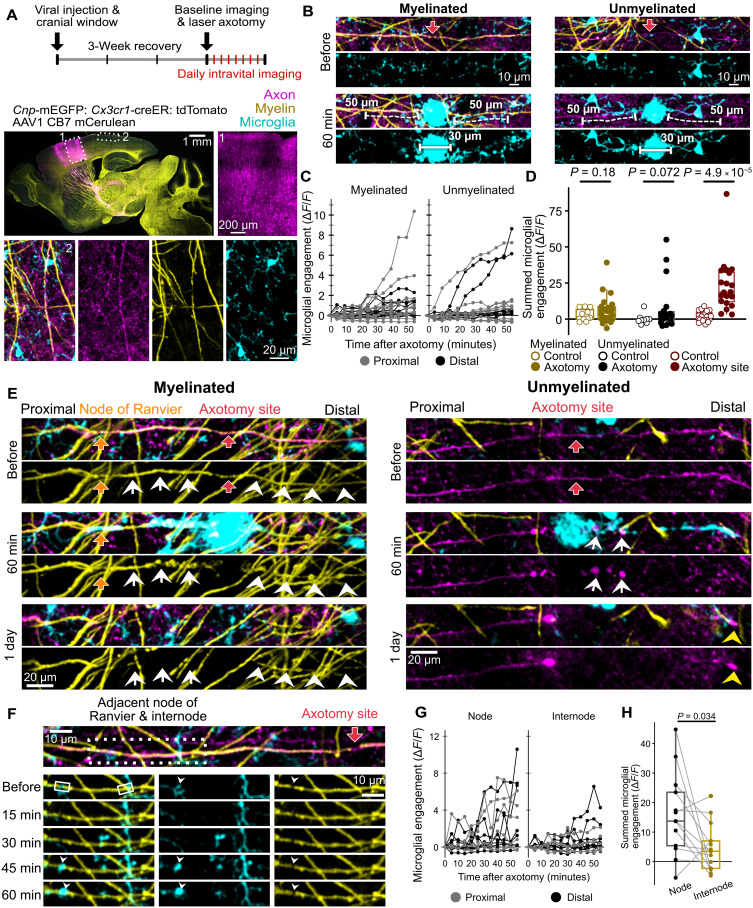
Microglia exhibit rare and precise engagement with axons acutely after axotomy. (**A**) Experimental timeline and strategy for triple fluorescent labeling. Box 1 shows viral labeling of cells in the motor cortex. Box 2 indicates the somatosensory cortex, which was imaged intravitally (bottom). (**B**) Acute microglial response to axotomy. Indicated 30-μm ROIs are associated with nonspecific microglial response [quantified in (D)]. Microglial engagement with injured axons was analyzed in indicated 50-μm ROIs [quantified in (C) and (D)]. (**C**) Traces of acute microglial engagement. (**D**) Summed microglial engagement in the hour following axotomy for myelinated axons, unmyelinated axons, and the region of the axon within the axotomy site, compared to controls (Welch’s *t* tests). (**E**) Image series showing robust microglia engagement with the proximal segment of a myelinated axon and the distal segment of an unmyelinated axon (white arrows). White arrowheads point to a de-axoned myelin sheath. For the unmyelinated axon, yellow arrowhead points to the retraction bulb 1 day after axotomy. (**F**) Proximal myelin sheath and node of Ranvier for an axotomized axon; the dotted box indicates the region shown in the image series below. White arrowheads point to microglia engagement at node of Ranvier, which was analyzed with 5-μm ROIs (solid line boxes). (**G**) Traces of acute microglial engagement at nodes and internodes. (**H**) Microglial engagement at nodes and internodes, summed in the first hour after axotomy (paired *t* test). For axotomized axons in (C) and (D), *n* = 22 myelinated and 18 unmyelinated axon segments from 20 axotomies in eight mice. For controls in (D), *n* = 11 myelinated and 9 unmyelinated axons from eight mice. For (H) and (G), *n* = 13 node-internode pairs from 10 axotomies from seven mice. Detailed statistical results in data S1.

Microglia are the resident phagocytes of the brain and are known to clear neuronal somas after apoptosis and myelin after oligodendrocyte death (*46*, *58*, *59*). Microglia are also thought to clear axons following Wallerian degeneration, but this has never been directly observed (*25*, *60*, *61*). Following our observation of myelination hindering axonal debris clearance after neuronal 2Phatal, we asked whether microglia-mediated phagocytosis differed between myelinated and unmyelinated axons following axotomy. We also asked whether microglia engage with myelin sheaths of axotomized axons. To simultaneously visualize oligodendrocytes, microglia, and axons, we used a viral strategy to label axons with monomeric cerulean (mCerulean) in a triple transgenic mouse line expressing mEGFP in oligodendrocytes and tdTomato in microglia [*Cnp*-mEGFP: *Cx3cr1*-creER^+/−^: tdTomato] (Fig. 5A and fig. S5). Virus (AAV1-CB7-mCerulean) was injected into the motor cortex, so that with a cranial window over the somatosensory cortex, we visualized the axons of neurons projecting from the motor cortex through layer I of the somatosensory cortex. These axons were therefore called motor cortex projections (Fig. 5A and fig. S5). We then performed laser-mediated axotomies at nodes of Ranvier and acquired a 1-hour time series immediately after the axotomy, as well as daily follow-up imaging for 7 days.

First, we focused on microglia engagement within the first hour after axotomy. Microglia rapidly extend their processes to laser lesion sites due to spillage of cellular contents (*46*, *47*). This phenomenon was observed at the axotomy site and consistently had a diameter of 30 μm or less (Fig. 5B). Because this chemotactic response is associated with general tissue damage and is not specific to axon damage, we focused on microglia engagement with axons outside of this region (Fig. 5B). Therefore, microglia engagement was measured in 50-μm-length ROIs (5-pixel width) aligned with targeted axons, from 15 to 65 μm away from the axotomy site (Fig. 5B).

In the first hour after axotomy, microglia response varied from no engagement to a broad, robust engagement with the injured axon, for both distal and proximal axon segments (Fig. 5, C to E). However, overall, microglia engagement in the first hour after axotomy was not significantly different between axotomized and uninjured control axons, for both myelinated and unmyelinated axons (Fig. 5, C and D, and fig. S6, A and B; *n* = 22 axotomized myelinated, 11 control myelinated, 18 axotomized unmyelinated, and 9 control unmyelinated axon segments from eight mice). This contrasts the significant microglia engagement observed with the axon at the axotomy site (Fig. 5, B and D, and fig. S6, A and B; *n* = 20 axotomized and 20 control axon segments from eight mice). Curiously, robust engagement (summed Δ*F*/*F* > 20 in the first hour after axotomy) was observed for a small proportion of myelinated and unmyelinated axons (2 of 22 myelinated and 3 of 18 unmyelinated axon segments; Fig. 5, C to E). For myelinated axons, such robust engagement resulted in rapid loss of the axotomy-adjacent myelin sheath and underlying axon within 1 day (Fig. 5E, myelinated proximal). In clear contrast, minimal acute microglia engagement was observed with persistent de-axoned myelin sheaths, despite acute axon retraction (Fig. 5E, myelinated distal). Robust microglia engagement with an unmyelinated axon resulted in rapid loss of the axon, specifically within the region of engagement (Fig. 5E, unmeylinated distal).

We speculated that robust microglial engagement with axotomized, myelinated axons may be due to inadvertent damage to the myelin sheath. Therefore, we tested whether direct damage to the myelin sheath induces a robust microglia response and rapid myelin sheath clearance. We targeted axotomies to the myelin paranode, to replicate the myelin damage that may have inadvertently occurred previously. Axotomies targeting myelin paranodes resulted in a microglial response in some but not all instances (fig. S7, A, C, and D; *n* = 5 axotomies from three mice). However, we never observed clearance of the myelin sheath within 1 day of the paranode injury (fig. S7, A and E; *n* = 5 axotomies from three mice). Axotomies targeting the center of the myelin internode did induce rapid microglial engagement with the myelin sheath (fig. S7, B to D; *n* = 4 axotomies from three mice). In three of four axotomies, this resulted in clearance of the myelin sheath within 1 day of the internode injury (fig. S7, B and E; *n* = 4 axotomies from three mice). Therefore, direct damage to the myelin sheath can induce rapid microglial engagement and rapid myelin sheath clearance in an inconsistent manner that may depend on the location of the injury and the extent of direct damage to the myelin sheath.

Following axotomies at nodes of Ranvier, in instances where no robust, broad microglial engagement was observed, we noted some microglial engagement at adjacent nodes of Ranvier (Fig. 5F and fig. S8). To quantify this, we compared microglial engagement at axotomy-adjacent nodes of Ranvier to axotomy-adjacent myelin internodes (Fig. 5F). While microglial engagement was heterogeneous at nodes and internodes, specific microglial engagement occurred more frequently at nodes (Fig. 5G). Statistical comparison of summed microglial engagement in the first hour after axotomy revealed significantly higher microglial engagement at nodes compared to adjacent internodes (Fig. 5H and fig. S6C; *n* = 13 node-internode pairs from seven mice). Similar microglia engagement at nodes has been reported following axotomy in the spinal cord (*51*). In the previous study, microglial engagement at nodes proximal to the axotomy was described as protective and corresponded with reduced axon degeneration (*51*). Thus, we looked for a relationship between precise microglial engagement at proximal nodes and degeneration of proximal axons by measuring the distance between the axon end (or axotomy site) and the proximal node (fig. S8). We found that axons with microglia engagement (summed Δ*F*/*F* > 20 in the first hour after axotomy) at the proximal node retract to or past the proximal node (*n* = 3 axons; fig. S8, A, C, and E). In contrast, when microglia do not engage with the proximal node, the proximal axon exhibits only minor retraction (*n* = 2 axons; fig. S8, B, D, and E). Together, acute microglia responses to axotomy are rare, precise, and often correspond with acute clearance of myelin and/or axon.

### Myelination status does not affect axon degeneration or microglial engagement

**Fig. 6.**
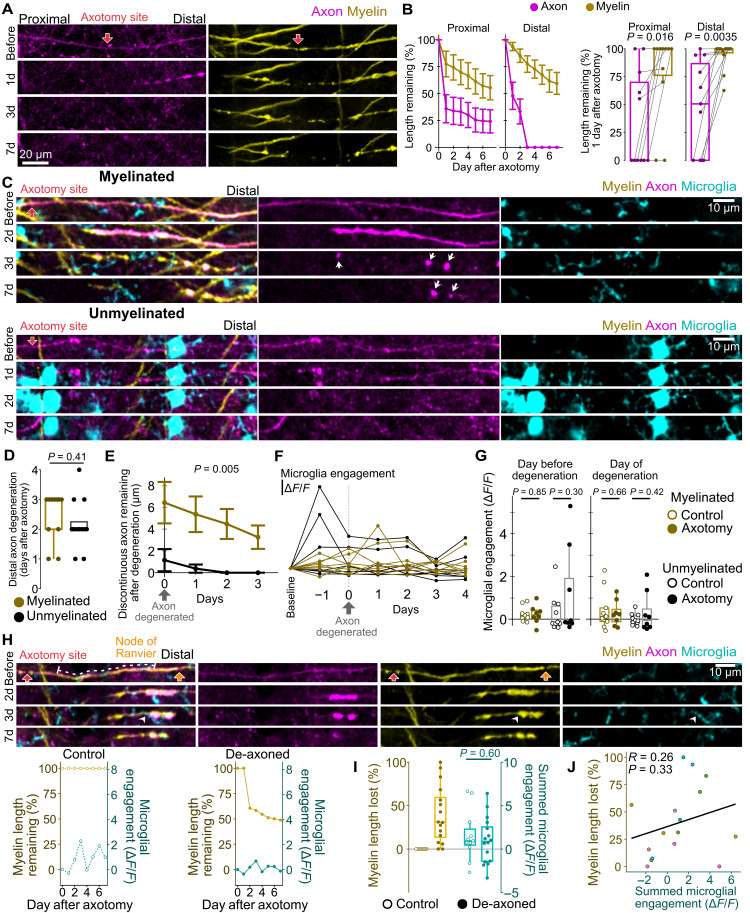
Microglia do not respond to axons undergoing Wallerian degeneration or de-axoned myelin sheaths. (**A**) De-axoned myelin persists after axotomy of motor cortex projections. Axon also shown in Fig. 5A. (**B**) Loss of axon and myelin throughout the first week [left, mean ± SEM, two-way repeated measures ANOVA, interaction terms *P* = 0.013 (proximal) and *P* = 0.004 (distal)] and in the first day (right, paired *t* tests) after axotomy (*n* = 11 axotomies from seven mice). (**C**) Microglia during Wallerian degeneration and discontinuous axon debris (white arrows). (**D**) Day of degeneration is the first day when the axon is no longer continuous (Welch’s *t* test). (**E**) Total length of discontinuous axon fragments after axon degeneration (two-way repeated measures ANOVA, shown *P* value comparing myelination status). (**F**) Microglial engagement during Wallerian degeneration of distal axon segments. (**G**) Microglial engagement the day before (left) and day of (right) degeneration compared to control axons (Welch’s *t* tests). (**H**) Microglial engagement (white arrowhead) with de-axoned myelin sheath and analyzed 50-μm ROIs (dotted line). On the bottom, myelin length remaining is overlaid with microglial engagement for a control sheath (left) and the shown de-axoned (right) myelin sheath. (**I**) Myelin length lost and summed microglial engagement in the week after axotomy for de-axoned myelin sheaths and controls (Welch’s *t* test). (**J**) Pearson correlation between myelin length lost and summed microglial engagement. Data points are colored by animal. For (D) and (E), *n* = 11 myelinated and 12 unmyelinated axons from eight mice. For axotomized axons in (F) and (G), *n* = 8 myelinated and 8 unmyelinated axon segments from seven mice. For controls in (G), *n* = 9 myelinated and 10 unmyelinated axons from seven mice. For (I) and (J), *n* = 16 de-axoned and 11 control myelin sheaths from seven mice. Detailed statistical results in data S1.

Consistent with PV+ axons, axotomy of motor cortex projections resulted in rapid degeneration of axons but persistence of de-axoned myelin (Fig. 6, A and B, and fig. S6D; *n* = 11 axotomies from seven mice). We asked whether the presence of myelin affects patterns of axon degeneration following axotomy. Local axon topography, including synaptic density, distance to axon termini, and proximity to branch points, reportedly correlates with degeneration (*44*, *53*). In our dataset, axon termini were not discernible within our image volume. To match the lack of synapses on myelinated axons, we selected unmyelinated axons that morphologically appeared to have few en passant synaptic boutons. In this analysis, axons did not have discernible branch points within the image volume. In addition, to compare local axon topography, we measured axon width and found no difference between targeted myelinated and unmyelinated axons (fig. S9A; *n* = 11 myelinated and 12 unmyelinated axons from eight mice). Therefore, other than myelination status, we found no major differences between myelinated and unmyelinated axons that might affect degeneration.

Proximal to the axotomy site, the distance retracted was similar between myelinated and unmyelinated axon stumps, with the most length lost in the first day after axotomy (fig. S9, B and C; *n* = 10 myelinated and 11 unmyelinated axons from eight mice). In addition, no successful regeneration was observed for either myelinated or unmyelinated axons, although two (one myelinated, one unmyelinated) transient sprouting events were observed (fig. S9D). Distal to the axotomy site, axons underwent Wallerian degeneration between 1 and 4 days after axotomy, with no difference in this timing between myelinated and unmyelinated axons (Fig. 6, C and D, and fig. S6E; *n* = 11 myelinated and 12 unmyelinated axons from eight mice). However, consistent with 2Phatal results, axon debris was cleared less efficiently from myelinated axons, as discontinuous axon fragments were observed up to 1 week after axotomy (Fig. 6, C and E, and fig. S10; *n* = 11 myelinated and 12 unmyelinated axons from eight mice).

Next, we determined whether microglia engage with axons following axotomy, and whether variability in microglial engagement could account for the difference in axon debris clearance between myelinated and unmyelinated axons. The acute microglial response at the axotomy site was consistently cleared within 1 day of axotomy; however, microglia occasionally migrated to the site of injury and resided there in follow-up time points (Fig. 6C). Therefore, to measure microglial engagement specifically with transected axons, 50-μm ROIs were aligned with remaining regions of axon at least 15 μm away from the axotomy site (fig. S11, A to C). In the week after axotomy, we found that microglia engagement did not vary between proximal axon stumps and uninjured control axons, for both myelinated and unmyelinated axons (fig. S11; *n* = 7 axotomized myelinated, 10 control myelinated, 9 axotomized unmyelinated, and 11 control unmyelinated from eight mice). Unexpectedly, a similar lack of microglial engagement was observed during Wallerian degeneration (Fig. 6, C, F, and G, and fig. S10). On the day before and the day of Wallerian degeneration, we found that microglial engagement with degenerating axons was no different from microglial engagement with uninjured control axons, regardless of myelination status (Fig. 6, C, F, and G, and fig. S10; *n* = 8 axotomized myelinated, 9 control myelinated, 8 axotomized unmyelinated, and 10 control unmyelinated axons from seven mice). These results demonstrate that myelination minimally affects axon degeneration following axotomy and that microglia exhibit an unexpected lack of engagement with injured axons and axons undergoing Wallerian degeneration.

### Microglia do not respond to de-axoned myelin

While de-axoned myelin sheaths are remarkably persistent, they do gradually lose length following axotomy (Fig. 6, A to C and H, and fig. S12). Previous work in our laboratory captured microglia-mediated clearance of myelin sheaths following oligodendrocyte death (*59*). Therefore, we asked whether microglia contribute to the clearance of de-axoned myelin. Upon measuring microglial engagement with de-axoned myelin, we observed only minor fluctuations in microglial fluorescence, corresponding to brief sampling events (Fig. 6H and fig. S12). These sampling events were not associated with obvious loss in de-axoned myelin length, although de-axoned myelin sheaths did gradually lose length in the week after axotomy (Fig. 6, H and I, and fig. S12). In addition, microglia sampling of de-axoned myelin sheaths was not statistically different from microglia sampling of control myelin sheaths on uninjured axons (Fig. 6, H and I, and fig. S12; *n* = 16 de-axoned and 11 control myelin sheaths from seven mice). Furthermore, a Pearson correlation revealed no relationship between myelin length lost and microglial engagement (Fig. 6J). Therefore, we found no microglial response or engagement associated with the slow and progressive shrinkage of de-axoned myelin.

## DISCUSSION

Following axotomy, the process through which axons degenerate is well characterized and highly conserved across injury modalities (*62*). However, relatively little is known about how myelin or oligodendrocytes respond to or are affected by axon degeneration. Here, with longitudinal intravital imaging and laser-induced axon loss, we discover an unexpected persistence of de-axoned myelin. Persistence of de-axoned myelin sheaths is observed after Wallerian degeneration distal to an axotomy, after axon retraction proximal to an axotomy, and after neuronal apoptosis. De-axoned myelin is found after loss of PV+ axons and axon projections from the motor cortex. Therefore, persistence of de-axoned myelin occurs across injury modalities and neuronal subtypes. Furthermore, axon loss had a minimal effect on local oligodendrocyte homeostasis, with only a brief pause in myelin sheath extension. Last, we discover an unexpectedly minor response from microglia in association with Wallerian degeneration or de-axoned myelin. Together, these data highlight the stability of myelin and myelinating oligodendrocytes following axon injury.

To date, axotomy studies in the CNS have mostly focused on axon degeneration and regeneration (*44*, *45*, *50*–*54*). While these studies include axons of the spinal cord, which are commonly myelinated, myelin was either not imaged (*50*, *53*) or axotomy-adjacent myelin sheaths were not formally analyzed (*51*, *52*). Here, with dual fluorescent labeling of axons and myelin, we targeted nodes of Ranvier to transect the axon while minimizing damage to the myelin. We found remarkable persistence of myelin sheaths long after the loss of their axons. In our data, the rapid loss of myelin sheaths corresponded with uniquely robust microglial engagement acutely after axotomy. We speculated that accelerated myelin clearance may result from microglial responses to inadvertent myelin damage. However, when we directly damaged myelin paranodes, we never observed rapid clearance of myelin sheaths. Similarly, microglia did not always engage with myelin sheaths after paranode damage. Therefore, accidental damage to myelin paranodes does not explain robust microglial responses after axotomy at a node of Ranvier, and it remains unclear what causes these rare events. In contrast, direct damage to the center of the myelin internode did result in rapid clearance of myelin sheaths in most, but not all, cases. This is in line with previous work where myelin sheaths did not appear to persist after axotomy (*51*, *52*). Consistent with our results, de-axoned myelin has been described in human tissue collected after spinal cord injuries or stroke (*41*, *42*). It is unknown how this persistent myelin affects recovery or health of the nervous system after injury. On one hand, myelin inhibits axon growth (*63*, *64*), and myelin debris can elicit an immune response capable of propagating further damage after injury (*65*). Alternatively, persistent myelin could serve as a barrier to isolate prodegenerative cues released by degenerating axons (*66*–*68*). More work is needed to explore these possibilities.

The impact of axon loss on oligodendrocyte homeostasis has been studied following broad injuries, including enucleation, optic nerve crush, or TBI (*33*–*35*, *39*, *40*). These studies have produced mixed conclusions, including oligodendrocyte death (*35*, *39*), oligodendrocyte quiescence (*39*, *40*), or survival of oligodendrocytes at normal densities (*34*, *40*). The variability in conclusions may be related to variability in age at the time of injury, injury modality, or methods for identifying oligodendrocytes. Myelin pathology, including demyelination, is common following TBI (*33*–*35*). However, with broad injuries, it is difficult to ascertain whether myelin abnormalities result from axon loss or direct damage to myelinating cells. Our analysis reveals the resilience of oligodendrocytes following axon injury and loss. Local to injuries, oligodendrocytes maintained a healthy morphology, and oligodendrogenesis occurred at a normal rate. In addition, local myelin sheath plasticity appeared mostly normal, with a reduction in extending myelin sheaths limited to just the first week after injury. This temporary effect in myelin sheath plasticity could reflect an oligodendrocyte response to loss of an axon or it could be a direct response to signals released from the axotomy-associated tissue damage. We observed no abnormal myelin retraction or other disruption to myelin sheaths wrapping uninjured axons, indicating that oligodendrocytes maintain normal homeostatic function following axon loss and that single axon loss alone does not produce myelin pathology on intact axons. It remains unknown whether an oligodendrocyte maintains similar stability following the loss of more than one of its ensheathed axons. Future work scaling up the number of damaged axons could investigate persistence of de-axoned myelin following broader axon loss.

Effective clearance of axon and myelin debris after injury is thought to be important for healing and regeneration (*63*, *64*, *69*–*72*). In the PNS, myelinating Schwann cells actively contribute to the clearance of axon debris through constriction of myelin to accelerate axon fragmentation and phagocytosis (*25*–*29*). In contrast, in the CNS, myelinating oligodendrocytes have not been described to exhibit any of these functions and are unable to facilitate axon debris clearance in vitro (*26*, *73*). Our data show axon debris lingering under myelin for a week or longer after the injury, consistent with oligodendrocytes lacking meaningful phagocytic function. We do observe myelin swellings around axon fragments, possibly reminiscent of constricting myelin reported in Schwann cells (*26*). However, we cannot differentiate myelin constricting to create axon fragments or myelin conforming to the shape of underlying fragments. Furthermore, in our data, myelination status was unimportant for the timing of axon degeneration, supporting the idea that myelin in the CNS does not facilitate axon fragmentation, degeneration, or clearance after injury.

In the CNS, microglia are thought to mediate clearance of axon debris following axon transection and Wallerian degeneration (*25*, *61*). Microglia are known to respond immediately to focal injury and also phagocytose dying cells (*46*, *47*, *58*, *59*). Molecular signals including adenosine triphosphate (ATP), potassium, or phosphatidylserine are detected by microglia as indicators of cellular damage (*47*, *74*–*76*). Following an axon transection and a break in the plasma membrane, ATP and potassium diffuse into the extracellular space. Phosphatidylserine is externalized in the plasma membrane of damaged and stressed axons, most prominently on axonal spheroids (*68*, *77*). Therefore, we predicted that microglial engagement might be observed with axotomized axons, especially with unmyelinated axons, where the axon is directly accessible to microglia processes. Unexpectedly, microglial engagement with transected axons was rare. Acutely, microglial engagement was not different between axotomized and control axons, although we did observe robust engagement in a small proportion of axotomy trials. An explanation for heterogeneity in acute microglial response is unclear, but some possible variables could be timing to reseal the axonal plasma membrane, diffusion of spilled intracellular contents, damage to axonal organelles, or chance interactions with microglial processes. For some myelinated axons, we also observed precise microglial engagement at nodes of Ranvier acutely. Microglial engagement at nodes has been reported previously, in baseline conditions and following injury (*51*, *78*). This engagement can be regulated by potassium (*78*) or ATP (*51*) release and is reported to limit proximal axon retraction following axotomy in the spinal cord (*51*). From our data in the cortex, microglial engagement at proximal nodes corresponds with greater axon retraction, although more work is needed to confirm this pattern. Why our results contrast with previous findings may relate to selection of the axotomy site or regional heterogeneity in glial cell responses to injury. In addition, it is unclear why microglia would exhibit this pattern of engagement at unmyelinated regions of myelinated axons but no pattern of engagement with fully unmyelinated axons.

Throughout the week following axotomy, we observed no patterns of microglial engagement with damaged or degenerating axons. This finding contrasts the established idea that microglia phagocytose axons undergoing Wallerian degeneration (*39*, *60*, *61*). However, consistent with our data, microglial phagocytosis of degenerating axons has never directly been observed. Microglia are activated generally and express phagocytic genes after injuries, including TBI, nerve transection, and nerve crush (*79*–*83*). However, immunostaining experiments after injury have been unable to convincingly identify phagocytic microglial contact with degenerating axons (*80*, *81*). Rod microglia engage with damaged axons (*84*, *85*); however, one experiment found these contacts to not be phagocytic (*85*). Microglia contact with axons has been observed with electron microscopy after TBI, however, at time points consistent with secondary injury rather than degeneration of axons damaged directly by the TBI (*80*, *86*). One study linked axon degeneration after spinal cord injury to phagocytosis by infiltrating monocytes rather than resident microglia (*87*). In degenerative disease, evidence used to claim microglia phagocytose axons includes prevalence of transected axons in inflamed brain regions and increased microglia density around dying neurons (*88*, *89*). However, no direct contact between neurons and microglia was reported in these experiments. Intravital work directly shows microglia phagocytosing somas of dying neurons (*46*, *58*). Astrocytes and microglia both contribute to the clearance of neuronal processes (*58*). Thus, a role for astrocytes in clearing axons after Wallerian degeneration is an intriguing possibility.

After injury and axon degeneration in the PNS, myelin is lost rapidly through processes including autophagy (*90*), necroptosis (*63*), and phagocytosis by peripheral macrophages (*27*, *71*). Our laboratory previously demonstrated phagocytosis of myelin and oligodendrocytes by microglia during oligodendrocyte death and demyelination (*59*). Here, we observe no pattern of microglia engagement or phagocytosis with de-axoned myelin sheaths. Loss of de-axoned myelin occurred gradually in the weeks after axotomy, in stark contrast to the phagocytosis of myelin in a single day during demyelination (*59*). The slow nature in which de-axoned myelin sheaths are cleared could be reminiscent of cell-intrinsic autophagy rather than a phagocytic process to clear myelin debris. Oligodendrocytes continually recycle lipids and proteins through autophagy (*91*) and are suggested to adjust their autophagic flux in response to the extracellular environment (*92*). Markers of autophagy are reportedly increased in oligodendrocytes following spinal cord injury, and oligodendrocyte autophagy is linked to functional recovery from injury (*93*, *94*). More work is needed to understand oligodendrocyte autophagy following axon injury.

In our study, we used laser-induced axotomy to cause precise injury of targeted axons. Care was taken to minimize damage in the surrounding cellular environment during axotomies; however, with such laser-induced injuries, nonspecific damage is unavoidable. Still, the study presented here produced insight into glial cell responses to axon injury and degeneration. It is unknown whether glial cell responses are dependent on the scale of the injury and whether our findings hold true for broader axon loss. In addition, while we find no evidence of microglial engagement during Wallerian degeneration, we cannot exclude the possibility that microglia-mediated phagocytosis of axons is too rapid to capture with daily imaging. In previous work from our laboratory, daily imaging sufficiently captured microglia engagement and phagocytosis of myelin sheaths during demyelination (*59*). However, phagocytosis of axons by microglia may occur on a shorter timescale, and therefore, temporal resolution is a limitation to this study.

Together, we show persistence of myelin sheaths following axon loss across injury modalities and neuronal subtypes. These findings highlight the resilience of oligodendrocytes and demonstrate that axon injury alone is not sufficient to induce myelin pathology. Furthermore, we find no pattern of microglial engagement associated with axon injury, Wallerian degeneration, and shrinkage of de-axoned myelin sheaths. Future studies focused on the clearance of axon debris and de-axoned myelin sheaths will broaden our understanding of neuroglia interactions and responses to injury.

## MATERIALS AND METHODS

### Animals

All animal procedures were approved by the Institutional Animal Care and Use Committee at Dartmouth College. Mice were group housed (two to four animals per cage) in a temperature (22°C) and humidity controlled (30 to 70% relative humidity) animal vivarium with a 12-hour light-dark cycle and free access to food and water. *Pv*-cre (*95*) [The Jackson Laboratory (JAX), strain #008069], floxed tdTomato Ai9 (*96*) (JAX, strain #007909), *Cnp*-mEGFP (*97*) (JAX, strain #026105), and *Cx3cr1*-creER (*98*) (strain #020940) were purchased from the Jackson Laboratory and crossed to generate dual or triple transgenic mice. All experiments were performed with male and female mice aged 2 to 6 months at the time of cranial window surgery. Animals were excluded from analysis if cranial window quality precluded confident axon and myelin tracing. For microglia engagement experiments, mice heterozygous for the cre-ER fusion protein were selected so that *Cx3cr1* expression was maintained. Cre expression was induced in *Cx3cr1*-creER mice at weaning by administering 0.05 ml of tamoxifen (20 mg/ml in corn oil) for 2 consecutive days.

### Surgery

Cranial windows were implanted above the left and right somatosensory cortices for intravital imaging. Animals were anesthetized by an intraperitoneal injection of ketamine (10 mg/kg) and xylazine (10 mg/kg). Fur on the head was shaved, and skin was sanitized with alcohol and betadine wipes. Skin and connective tissue over the skull were removed. To the rostral portion of the skull, a nut was secured with glue and dental cement to facilitate head immobilization for repeated imaging. For viral labeling of motor cortex axons, stereotaxic injection was performed at this point (viral delivery described below). A craniotomy was performed with a high-speed drill to remove a circular, 3-mm-diameter section of skull. The underlying dura was also removed. For 2Phatal experiments, Hoechst 33342 nuclear dye [diluted to 0.1 mg/ml in sterile phosphate-buffered saline (PBS)] was applied topically to the cortical surface. The cortex was then covered with a round piece of no. 0 cover glass, and slight pressure was maintained as glue and dental cement were used to secure the cover glass in place. Carprofen (5 mg/kg) was administered subcutaneously before, immediately after, 24 hours after, and 48 hours after surgery.

### Viral delivery

To sparsely label PV+ interneurons for 2phatal experiments, PHP.eB FLEX-tdTomato (*99*) [titer 1.5 × 10^13^ genome copies (GC)/ml] was diluted 1:500 in PBS. *Cnp*-mEGFP: *Pv*-cre mice were anesthetized with isoflurane (3% for induction), and 100 μl was delivered via intravenous retro-orbital injection 3 weeks before 2Phatal. PHP.eB-FLEX-tdTomato was a gift from E. Boyden (Addgene, viral prep #28306-PHPeB; http://n2t.net/addgene:28306; RRID:Addgene_28306).

To fluorescently label axons with mCerulean, AAV1 CB7-mCerulean (titer 3.5 × 10^13^ GC/ml) was diluted 1:10 in PBS. Mice were prepped as described for cranial window surgery. A small hole was drilled in the skull, and a micromanipulator was used to navigate to the injection target. A total of 0.5 μl of diluted virus was injected with a glass micropipette into the motor cortex (Anterior-Posterior: +1.0 mm, Medial-Lateral: +1.0 mm, Dorsal-Ventral: −0.6 mm). After injection, cranial window surgery proceeded as described above. AAV1.CB7.CI.mCerulean.WPRE.RBG was a gift from J. M. Wilson (Addgene, plasmid #105557; http://n2t.net/addgene:105557; RRID:Addgene_105557).

### Intravital imaging

Mice were anesthetized with isoflurane (3% for induction, 1.5% during the imaging session) and head-fixed using the nut placed during the cranial window surgery. For baseline and follow-up imaging in axotomy experiments with *Cnp*-mEGFP: *Pv*-cre: tdTomato mice and SCoRe, images were acquired with an upright laser scanning confocal microscope (Leica SP8 with LasX software) equipped with a 20× water immersion objective [Leica; numerical aperture (NA), 1.0]. SCoRe (*49*, *100*) images were acquired by overlaying reflection produced by low-power 448-, 488-, 552-, and 638-nm lasers. Fluorescence images were acquired using the 488-nm laser to excite mEGFP and the 552-nm laser to excite tdTomato. SCoRe and fluorescence images were acquired sequentially between frames. All images were acquired as z-stacks with a step size set to 1.5 μm. Baseline and follow-up images were acquired at two positions in each window: axotomy and control positions.

For 2Phatal and microglia engagement experiments, images were acquired with a two-photon microscope (Bruker with Prairieview Software) equipped with an InSight X3 femtosecond pulsed laser (Spectra Physics) and a 20× water immersion objective (Zeiss; NA, 1.0). For 2Phatal experiments, fluorescence images were acquired using a 775-nm laser to excite Hoechst nuclear dye, a 920-nm laser to excite mEGFP, and a 1040-nm laser to excite tdTomato. In microglia engagement experiments, fluorescence images were acquired using an 820-nm laser to excite mCerulean, a 1040-nm laser to excite tdTomato, and a 950-nm laser to excite mEGFP without exciting mCerulean. In microglia engagement experiments, a time series was initiated immediately after axotomy to acquire a z-stack every 5 min for 1 hour. All images were acquired as z-stacks with a step size set to 1.5 μm. In 2Phatal experiments, images were acquired at baseline, 1, 7, 14, and 30 days after 2Phatal, in one to three 2Phatal positions in addition to one control position at baseline and 30 days after 2Phatal, per each cranial window. In microglia engagement experiments, images were acquired in two positions (one myelinated, one unmyelinated) per cranial window.

### Laser-induced axotomy

Laser axotomies were performed at least 21 days after cranial window surgery. All targeted axons were in layer I of the somatosensory cortex. Using baseline images, targeted myelinated axons were selected for having clear nodes of Ranvier or breaks in myelin, in addition to at least 65 μm of clear length on either side of the axotomy site. In experiments with *Pv*-cre:tdTomato mice, six axotomies targeted nodes of Ranvier (1- to 2-μm break in *Cnp*-mEGFP signal), and three axotomies targeted 28.6-, 45.6-, or 22.1-μm breaks in myelin. In experiments with viral labeling of motor cortex projecting axons, all axotomies targeted nodes of Ranvier (1- to 2-μm break in *Cnp*-mEGFP signal). Targeted unmyelinated axons were selected for having at least 100 μm of clear, unmyelinated length on either side of the axotomy site and morphologically few en passant boutons. Clear axon length can be defined as not precluded by vasculature, movement in z, edge of window, and clearly distinguished from neighboring axons. All laser axotomies were performed with a 2Photon microscope. An 8 by 12 pixel (4.27 x 6.4 μm) ROI was drawn at the target axotomy site, long side perpendicular to the axon. Axotomy was induced with ~8-s exposure to an ~75-mW laser, with the pixel dwell time set to 100 μs. To avoid multiple transections of the same axon, transections were limited to one myelinated and one unmyelinated or one paranode and one center of internode (when applicable) axon per cranial window. When applicable, images at control positions were taken at least 750 μm away from the axotomy site. When two axons were transected in the same window (one myelinated and one unmyelinated or one paranode and one center of internode), imaging positions for each targeted axon were at least 750 μm apart.

### 2Phatal

2Phatal was induced 1 day after cranial window surgery to ensure sufficient nuclear labeling. tdTomato expressing PV+ interneurons with Hoechst nuclear labeling were identified in layer II/III of the somatosensory cortex. All targeted neurons were between 120 and 250 μm below the pial surface. Using a 2Photon microscope, a 15 by 15 pixel ROI was centered on the targeted nucleus and exposed to 5.95 s (200 scans) of 775-nm laser, with a dwell time of 100 μs. One to three cells were targeted per cranial window.

### Sagittal brain slice preparation and imaging

After completion of longitudinal imaging for microglia engagement experiments, mice were anesthetized with ketamine (100 mg/kg) and xylazine (10 mg/kg) and perfused with 4% paraformaldehyde in PBS. Brains were dissected out and postfixed in 4% paraformaldehyde overnight at 4°C and then stored in PBS at 4°C until sectioning. Using a vibrating microtome (Leica, VT1000S), brains were sliced into 100-μm-thick sagittal sections. After slicing, brains were immediately mounted or stored in cryostorage solution [1% polyvinylpyrrolidone, 30% sucrose, 30% ethylene glycol, and 0.2 M sodium phosphate buffer (pH 7.4)] at −20°C.

Whole sagittal brain sections were imaged using tile scanning on an upright laser scanning confocal microscope (Leica SP8 with LasX software) equipped with a 10× air objective (Leica; NA, 0.4). mCerulean, EGFP, and tdTomato were excited using 448-, 488-, and 552-nm lasers, respectively.

### Quantification of axon and myelin sheath degeneration

To analyze structural change to axons and myelin sheaths after axotomy, the segmented line tool in Fiji was used to trace the targeted axon in the regions proximal and distal to the axotomy site. Axon segments were classified as proximal or distal depending on their pattern of degeneration. We cannot know for certain where neuronal somas and axon terminals are; however, our classification of proximal and distal is consistent with previous characterization of axon degeneration after axotomy (*44*, *45*, *50*)*.* Traces were saved and aligned in follow-up images using other myelin sheaths or axons as landmarks. These traces and distance along the trace were used to identify ROIs for analyses. To measure degeneration in the region corresponding to the axotomy-adjacent internode, measurements of length were recorded in the 50 μm adjacent to the lesion site. To measure degeneration in the region corresponding to the second proximal or distal internode, measurements of length were recorded in the 50 μm region beyond the next proximal or distal node of Ranvier. Transected axons form prominent retraction bulbs (*50*, *101*–*104*). The length of the axon remaining was measured to the tip of the retraction bulb. The length of myelin remaining was measured as the length of EGFP signal, accounting for breaks in signal >1.5 μm. In both confocal and 2Photon data, within a single myelin internode, breaks between bright pieces of myelin appear after axon loss. In 2Photon data, these bright fluorescent spots were connected by thin, dimly fluorescent myelin, thus appearing to be one continuous internode or de-axoned myelin sheath. However, such connections could not be visualized in confocal data, likely due to technical limitations. Therefore, measurements of myelin length in experiments using confocal microscopy are likely underestimations of remaining myelin length. When the axotomy site was a node of Ranvier, the axon was fully myelinated in 50-μm ROIs; however, this was not the case when the axotomy site was a longer break in myelin. Therefore, the length remaining within each 50-μm ROI is reported as a percentage to normalize for myelin length present before the axotomy. To measure SCoRe, reflection from each wavelength (448, 488, 552, and 638 nm) was merged into a single channel, and length was measured, accounting for breaks in reflectance signal >1.5 μm. SCoRe is reported as a ratio to EGFP, indicating the degree of myelin compaction (*49*, *55*, *105*).

Determination of whether the proximal axon retracted past the next proximal node of Ranvier was done with axons that had a discernible proximal node of Ranvier within the image volume. Similarly, analyses of second distal and second proximal internodes were performed with axons that had clear second distal and/or second proximal internodes within the image volume (>50 μm beyond the node of Ranvier proximal or distal to the axotomy site). The Fiji plugin SNT (*106*) was used for visualization of the traced axon, myelin, and SCoRe. Categorization of sheath with intact axon, sheath with axon debris, sheath with no axon, or axon and sheath both cleared was determined from the entire axotomy-adjacent internode, beyond the 50-μm ROI used in other analyses.

In 2Phatal experiments, analyzed axons were connected to a targeted neuron soma, had morphology reminiscent of en passant synapses (beads on a string), and had regions of myelination. The Fiji plugin SNT (*106*) was used to trace the axon of targeted neurons and subsequently identify associated myelin sheaths. Myelin sheaths approximately parallel to the pial surface were selected for analyses of myelin sheath length and axon debris. An eight-optical section max projection was created for each myelin sheath of interest. Myelin sheaths were traced and measured with the segmented line tool in Fiji. To analyze clearance of axon debris, the myelin trace (5-pixel width) at baseline was saved and aligned with the myelin sheath 1 day after 2Phatal. A line segment of equal length (5-pixel width) was traced on the adjacent unmyelinated region at baseline, saved, and aligned in the image acquired 1 day after 2Phatal. In each position, an additional line segment used for normalization of fluorescence (5-pixel width) was drawn to intersect both stably fluorescent and nonfluorescent regions. Plot profiles were used to record gray values for the tdTomato channel from each line segment. For each image, gray values were normalized by setting the minimum gray value to 0 and the maximum gray value to 1. Axon debris clearance is reported as the fold change in fluorescence from baseline to 1 day after 2Phatal.

### Quantification of axon width

Axon width was measured using full width at half maximum. For each targeted axon, three-line segments were drawn perpendicular to the axon, and plot profiles were recorded in the cerulean channel. Plot profiles were normalized by setting the minimum to 0 and the maximum to 1. The width at which the fluorescent signal peak was 0.5 and above was recorded for each line segment. The width measured from the three-line segments was averaged to estimate axon width.

### Quantification of oligodendrocyte homeostasis

To analyze oligodendrocyte homeostasis after axotomy, 11 optical slice max projections were made, centered at the axotomy site. To exclude the targeted axon and associated sheath, 100 μm by 150 μm ROIs were cropped on either side of the targeted axon, with the long side parallel to the targeted axon. Cropped images were excluded if vasculature limited visualization of fluorescent structures. Two ROIs of the same size were also cropped from images of control positions. The analysis of oligodendrocyte homeostasis was performed while blind to injury condition. Oligodendrocyte somas were identified, counted, and reported as a change in density from the baseline. To measure new myelin sheath generation, 15 myelin sheaths were selected at random in each ROI in the 14-day image. Sheaths were classified as stable if they were present in the 0-day image or classified as new if they were not present in the 0-day image. To measure myelin sheath plasticity, ends of six myelin sheaths were selected at random in each cropped image in the before time point, and their length was measured from an intersection with another myelin sheath, using the straight-line tool in Fiji. Sheath length change is reported as a change in length from the baseline image. An individual sheath was determined to be stable or plastic (extending or retracting) if the absolute value of its length change exceeded 1 standard deviation for length changes in that week (baseline to week 1 or week 1 to week 2).

To analyze oligodendrocyte homeostasis after 2Phatal, 21 optical slice max projections were made centered at the targeted neuron. A 150 μm by 150 μm ROI was then centered at the targeted neuron. ROIs of the same size were also drawn in control positions. Cropped ROIs were saved with the EGFP channel only, and analysis was performed while blind to injury condition. Oligodendrocyte somas were identified, counted, and reported as a change in density from the baseline.

### Quantification of microglial engagement

Time-series image volumes acquired acutely after axotomy were three-dimensional drift corrected based on the EGFP channel in Fiji. To measure microglia engagement with transected axons in acute time-series data and later follow-up images, eight-optical slice maximum projections were made centered on the targeted axon. In each image volume, uninjured control axons were identified in the same optical z-plane as the targeted axon, 30 to 150 μm away from the axotomy site. Control unmyelinated axons were identified in the cerulean channel, and control myelinated axons were identified in the EGFP channel. The segmented line tool in Fiji was used to trace targeted axons. Landmarks (other axons and myelin) were used to align the trace in follow-up images. In each imaging position, an additional normalization segment was drawn intersecting the targeted axon, stably fluorescent, and stably nonfluorescent regions and aligned at each time point. Line segment ROIs were set to a width of 5 pixels, and a microglial fluorescence gray value was recorded with plot profiles of the tdTomato channel. Fluorescence intensity was normalized by setting the minimum and maximum gray values for each image to 0 and 1, respectively. Microglial fluorescence is reported as the change in fluorescence at each time frame, relative to the first time frame, when microglia exhibit minimal response to the injury. Consistent with previous analyses, microglia engagement was measured in 50-μm ROIs. To confidently measure engagement with targeted axons and avoid measuring microglia response to nonspecific tissue damage from the laser injury, ROIs were shifted 15 μm distal or proximal from the injury site, thus avoiding the 30-μm region centered at the injury site. When axotomies targeted the center of an internode, microglia engagement was measured in two ROIs on either side of the axotomy site, summing to 50 μm. Microglial fluorescence in the 30-μm region of injury is reported as microglial engagement with the axotomy site. Microglial engagement at nodes of Ranvier was measured at a 5-μm ROI centered at a node and compared to a 5-μm ROI aligned on an internode of the same axon, 25 μm closer to the injury site. A threshold of 20 summed Δ*F*/*F* in all frames of the time series was used to categorize microglia as engaged or not (fig. S8).

Microglial fluorescence was measured in later time points to determine patterns of microglia engagement with proximal axon stumps, with distal axons during degeneration, and with de-axoned myelin sheaths. Microglial fluorescence intensity in daily follow-up images was normalized to the baseline image taken before the axon transection. Analyses continued to avoid the 30-μm-diameter region around the axotomy site to avoid any nonspecific microglia responses. Patterns of axon and myelin degeneration in microglial engagement experiments are thus reported from 50-μm ROIs starting 15 μm away from the axotomy site. For microglial engagement with proximal and distal axon stumps, if the axon retracted more than 15 μm in the first day after axotomy, 50-μm ROIs were aligned with the remaining axon. Analysis of microglial engagement at later time points is limited to axons with at least 50 μm of clear length remaining in the imaging field of view 1 day after axotomy. The day of degeneration for distal axons was defined as the first day in which no continuous axon is present.

### Statistical analysis

All statistical analyses were performed in R. No statistical methods were used to predetermine sample size. All data were assumed to have a normal distribution. For comparison of injured to control positions, experimenters were blind to condition for analyses. Two-way repeated measures analysis of variance (ANOVA) tested axon and myelin length remaining after injury, with time and structure (axon or myelin) treated as within-subject variables. Two-way repeated measures ANOVA tested change in oligodendrocyte soma density and change in myelin sheath length after axotomy, with time treated as a within-subject variable and position (axotomy or control) treated as a between-subject variable. Two-way repeated measures ANOVA tested the length of discontinuous axon length remaining after degeneration, with time treated as a within-subject variable and myelination status treated as a between-subject variable. Greenhouse-Geiser corrections was applied to two-way ANOVAs to address violations in sphericity when applicable (see data S1). One-way repeated measure ANOVA tested changes to the SCoRe:EGFP ratio. Paired *t* tests tested the difference between axon and myelin remaining at 1 day after injury, the difference in loss of axonal fluorescence after 2Phatal, and compared microglia engagement at nodes to internodes. Unpaired Welch’s *t* tests (two-sample, two-way) tested the differences in myelin sheath plasticity after axotomy, oligodendrocyte density after 2Phatal, day of distal axon degeneration, myelin length lost and microglia engagement with axotomized axons to uninjured controls. The relationship between myelin length lost and microglia engagement was assessed with a Pearson correlation coefficient.
