# Supplementary material for: Myelin sheaths persist for weeks following axon degeneration
Source: Sci Adv. 2026 Mar 4;12(10):eaeb2628. doi: 10.1126/sciadv.aeb2628 (PMC12959408; doi:10.1126/sciadv.aeb2628)
Supplement: Supplementary file 1 — Figs. S1 to S12 Legends for data S1 and S2 [file sciadv.aeb2628_sm.pdf]

Science Advances
AAAS

Supplementary Materials for

Myelin sheaths persist for weeks following axon degeneration

Megan E. Doty *et al.*

Corresponding author: Robert A. Hill, robert.hill@dartmouth.edu

Sci. Adv. **12**, eaeb2628 (2026)
DOI: 10.1126/sciadv.aeb2628

The PDF file includes:

Figs. S1 to S12
Legends for data S1 and S2

Other Supplementary Material for this manuscript includes the following:

Data S1 and S2

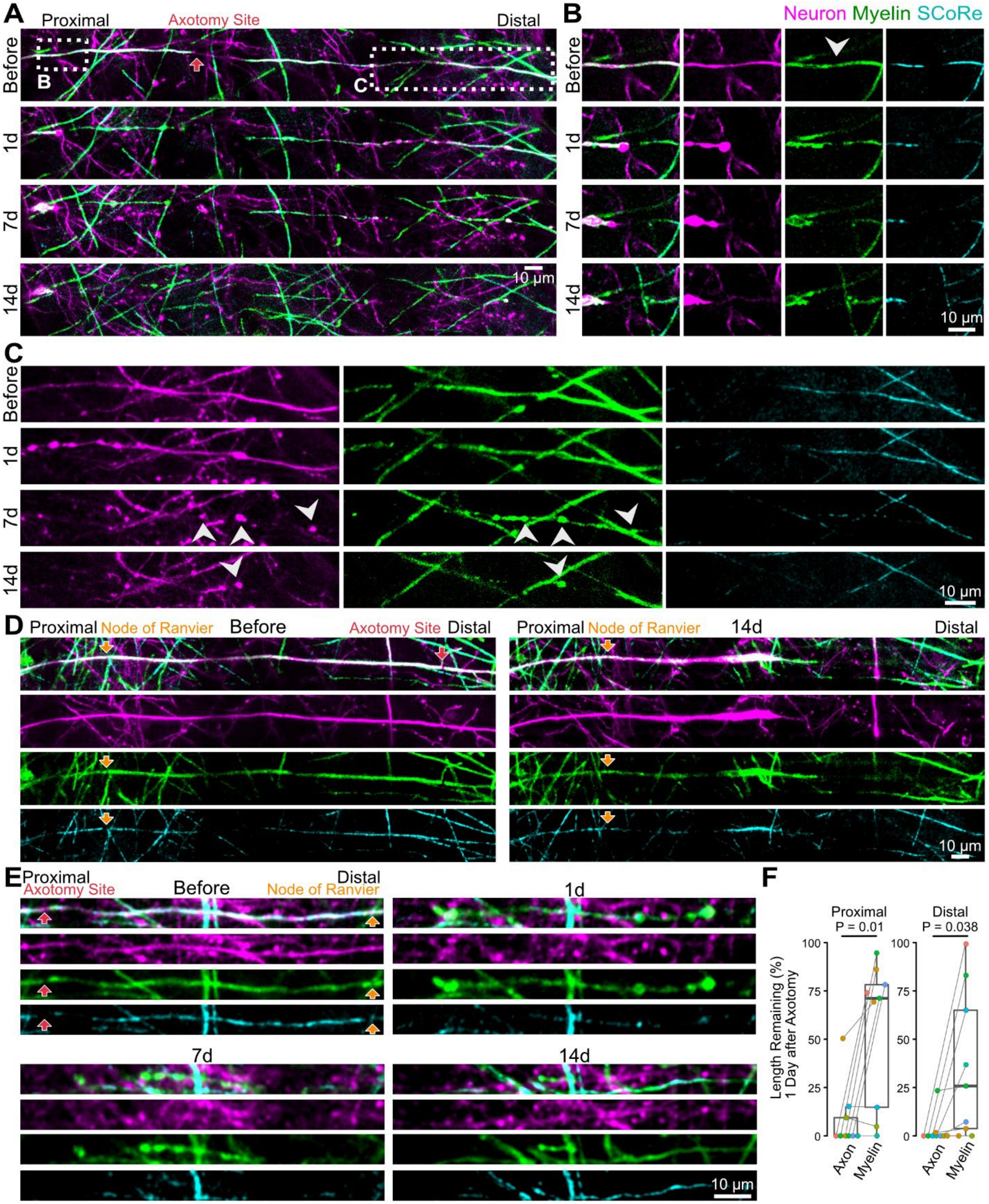

Figure S1: De-axoned myelin sheaths persist after axon degeneration. (A) Axon shown in Fig. 1, with myelin and SCoRe channels at all time points. **(B)** Proximal node of Ranvier as shown in Figure 1E, with myelin and SCoRe channels at all time points. Arrowhead points to node where adjacent myelin internodes appear to be linked by a paranodal bridge. **(C)** Second myelin sheath

distal to the axotomy site. Arrowheads point to axon debris associated with de-axoned myelin sheath after Wallerian degeneration. **(D)** Axotomized axon in which the proximal stump does not retract past the proximal node of Ranvier. This is the only proximal PV+ axon in our dataset with such limited retracted (see Fig. 1F). **(E)** Image series shows example of persistent de-axoned myelin sheath. **(F)** Fig. 2B, with data points colored by animal.

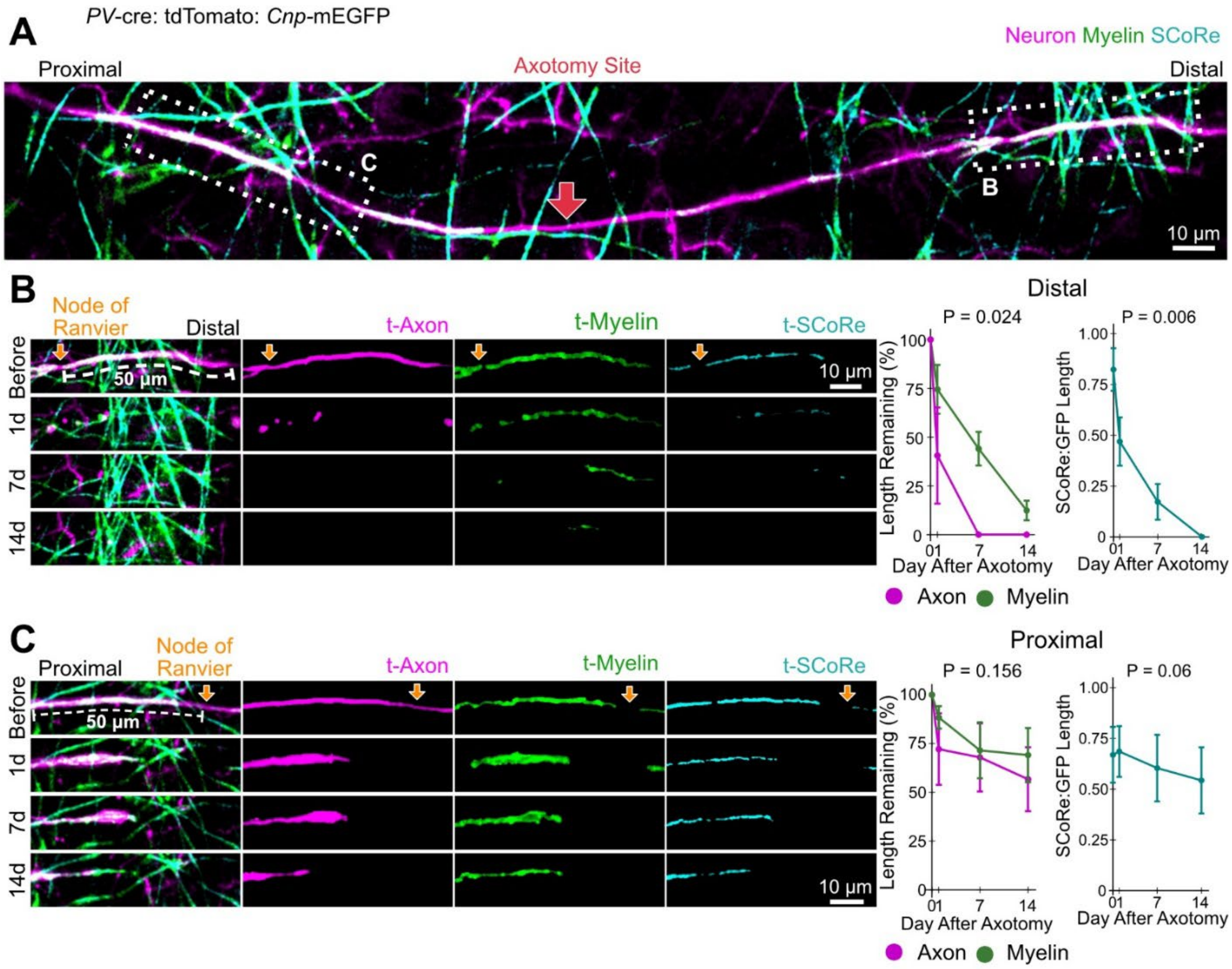

Figure S2: Secondary myelin internodes distal to axotomy persist following Wallerian degeneration and remain on proximal axon stumps. (A) Intravital image showing 2nd proximal and 2nd distal myelin sheaths relative to the axotomy site. **(B)** Merged intravital image, traced axon, traced myelin, and traced SCoRe of 2nd distal myelin sheath. Left graph represents the remaining axon and myelin as a percentage of baseline length remaining (mean ± SEM, n = 4 axotomies from 4 mice, significance determined from two-way repeated measures ANOVA, showing p-value for comparison of axon and myelin). Right graph represents SCoRe length as a ratio to GFP length (mean ± SEM, n = 3 persistent myelin sheaths from 3 mice, significance tested with repeated measures ANOVA). **(C)** Similar to (B) for 2nd proximal myelin sheath (n = 5 axotomies and 5 persistent myelin sheaths from 4 mice). Detailed statistical results in Data S1.

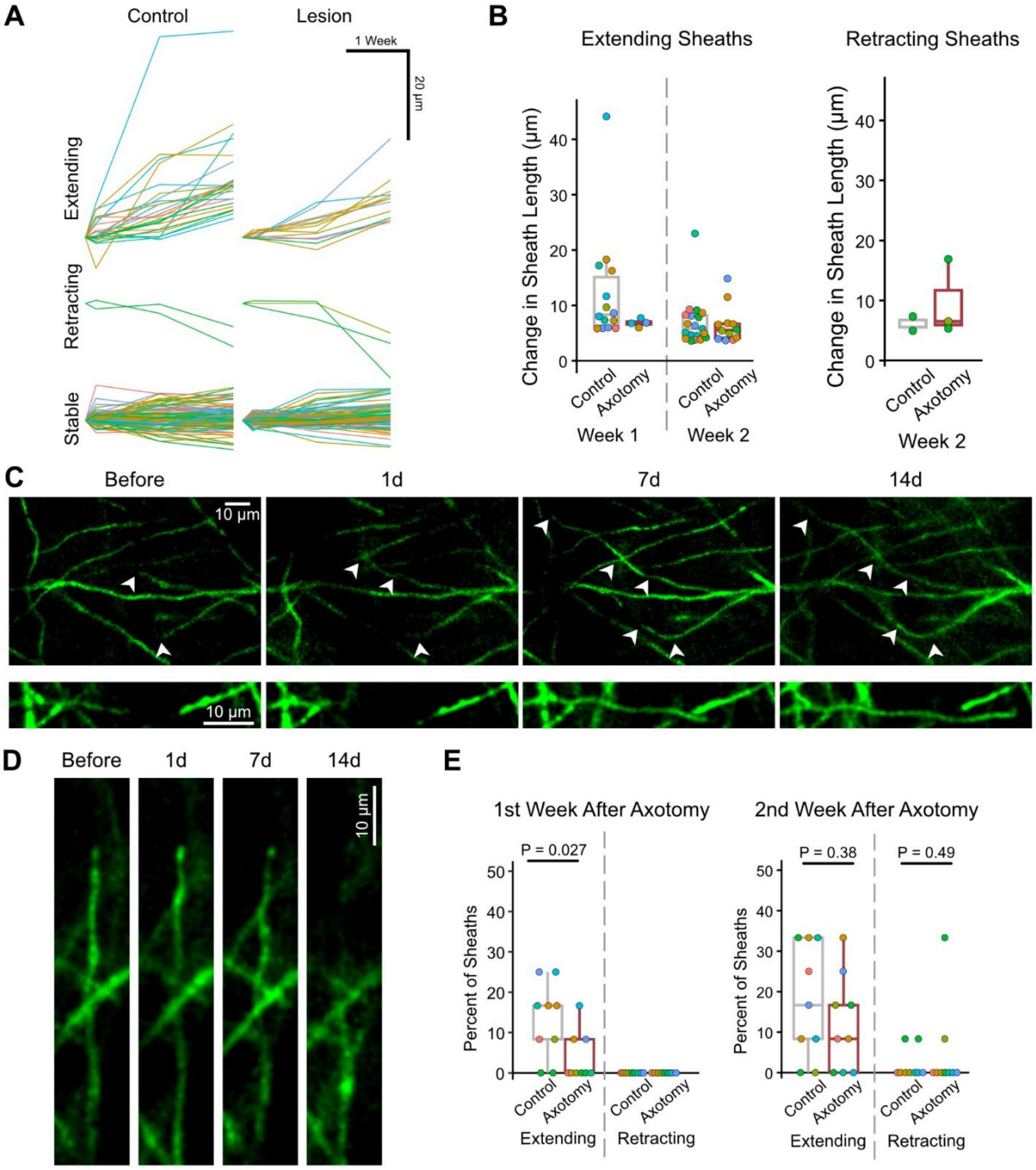

Figure S3: Reduced extension of myelin sheaths near axotomized axons. (A) Traces of length changes for all sheaths analyzed local to axotomy and in control positions. Traces are colored by animal. **(B)** Change in length for myelin sheaths classified as extending or retracting. Data points are colored by animal. **(C)** Examples of extending myelin sheaths. In the top row, arrowheads indicate the end of extending myelin sheaths. **(D)** Example of a retracting myelin sheath. **(E)** Fig. 3G with data points colored by animal.

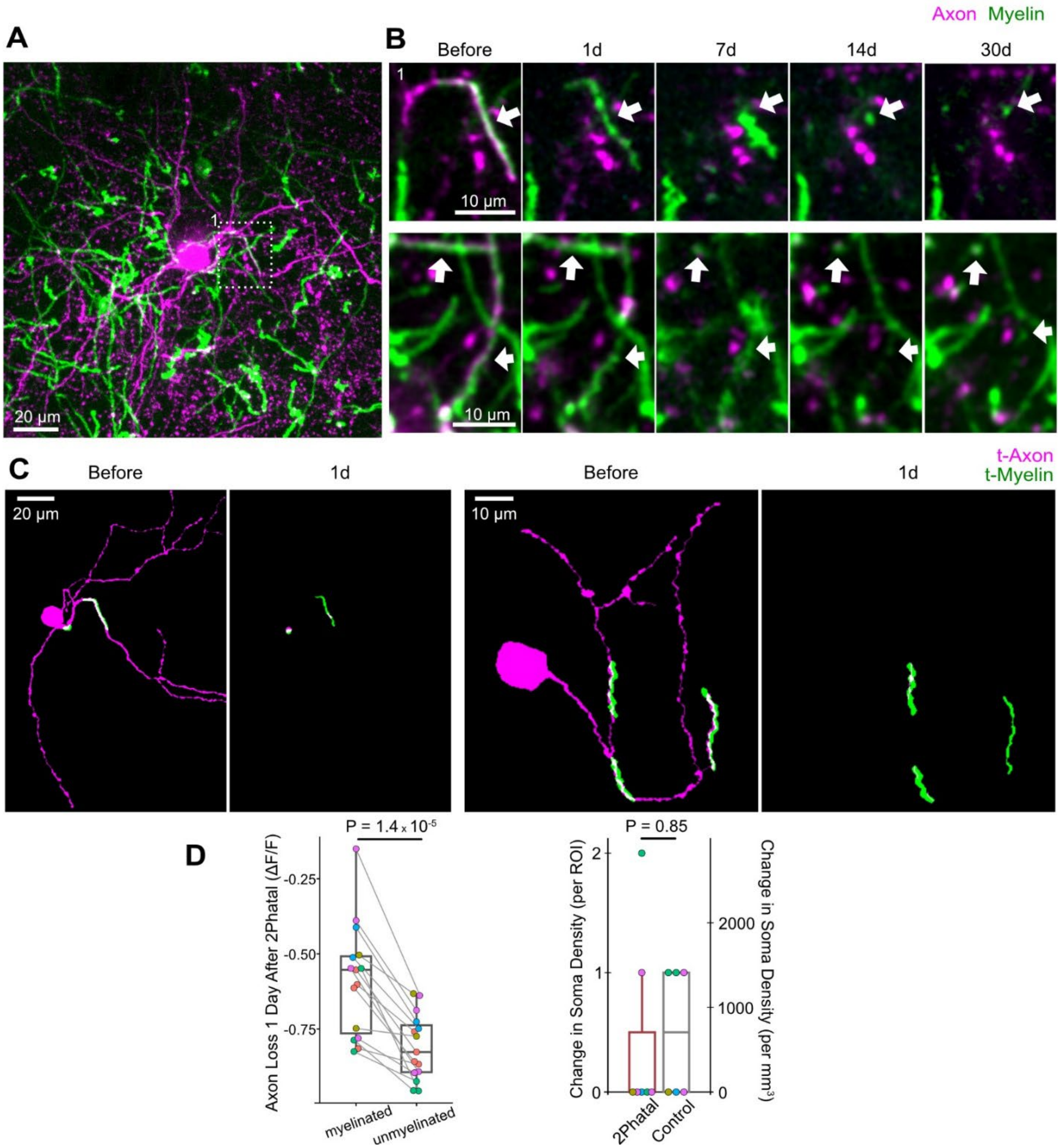

Figure S4: Myelin sheaths persist long after neuron death. (A) Image showing a PV+ cell that was targeted for 2Phatal. The boxed region labeled 1 is shown in the series in the top row of panel (B). **(B)** Image series showing myelin before and up to 30 days after 2Phatal. White arrows point to myelin ensheathing axons of targeted neurons. **(C)** Example traces of soma, axon, and associated myelin sheaths of targeted neurons, before and 1 day after 2Phatal. **(D)** Fig. 4G and I with data points colored by animal.

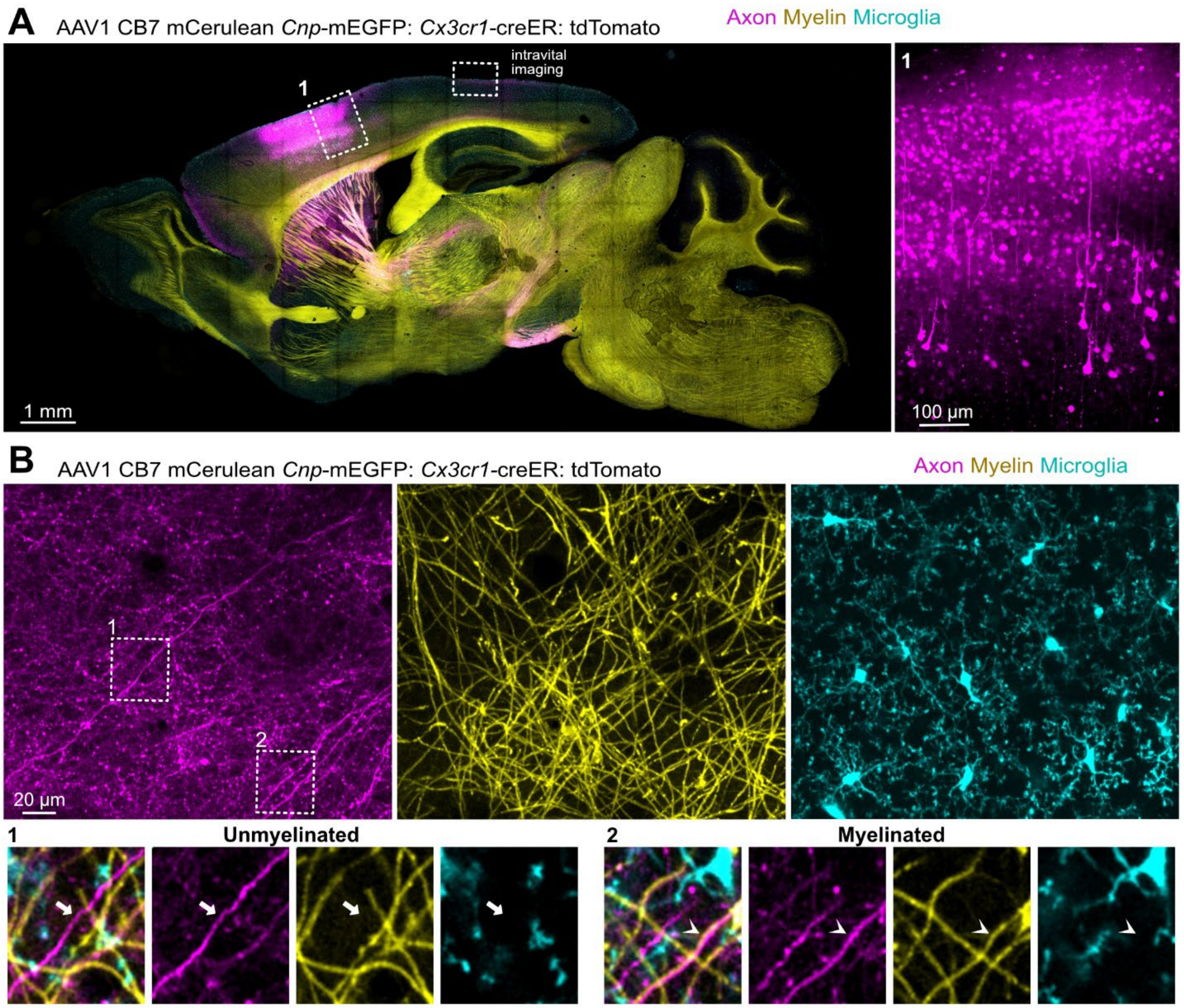

Figure S5: Strategy for triple fluorescent labeling of axons, myelin, and microglia. (A) Sagittal section of a brain with triple fluorescent labeling of myelinating oligodendrocytes (yellow), microglia (cyan), and motor cortex cells, including neurons projecting axons through the somatosensory cortex (magenta). Box 1 shows viral labeling of cells in the motor cortex. **(B)** Intravital images of the somatosensory cortex, including axons originating from the motor cortex (magenta), myelin (yellow), and microglia (cyan). In the region indicated by box 1, the white arrow identifies an unmyelinated axon. In the region indicated with box 2, the white arrowhead identifies a myelinated axon.

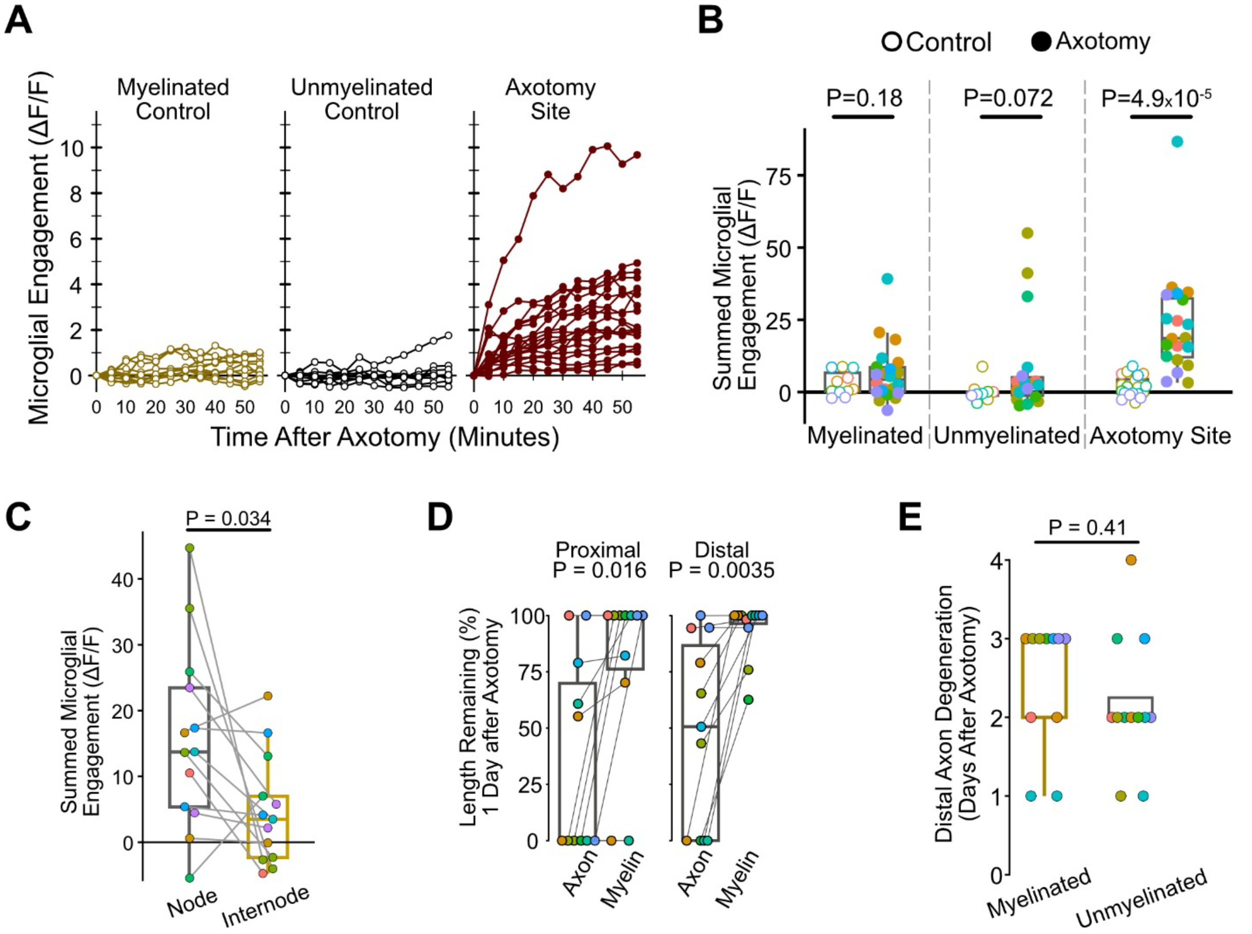

Figure S6: Additional quantification of microglia engagement. (A) Data associated with Figure 5D. Traces of microglia engagement with uninjured control axons (myelinated and unmyelinated, 50 µm ROIs) and the 30 µm region of axon centered on the axotomy site. **(B)** Figure 5D with data points colored by animal. **(C)** Figure 5H with data points colored by animal. **(D)** Figure 6B with data points colored by animal. **(E)** Figure 6D with data points colored by animal.

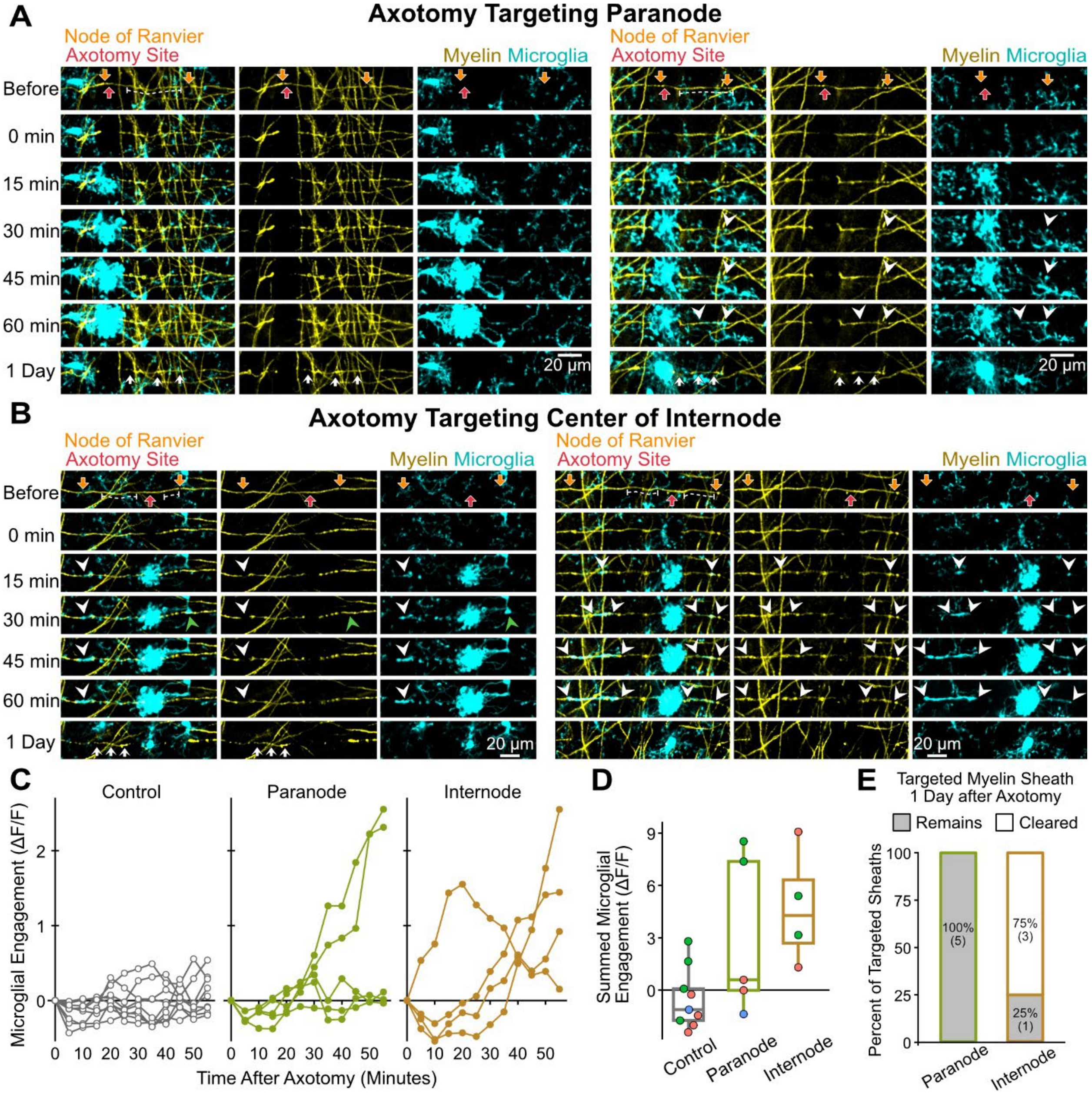

Figure S7: Microglia engagement following axotomy targeting the myelin sheath. (A) Image series showing microglia and myelin after axotomies targeting paranodes. Microglial engagement was analyzed in a 50 µm ROI (dashed white line). White arrowheads point to microglial engagement with the targeted myelin sheath. White arrows point to myelin sheaths remaining 1 day after axotomy. **(B)** Similar to (A) for axotomies targeting the center of internodes. ROIs on either side of the axotomy site add up to 50 µm. The green arrowhead points to transient microglial engagement with an untargeted myelin sheath. The targeted myelin sheath shown on the right is cleared 1 day after axotomy. **(C)** Microglial engagement with control myelin sheaths and myelin sheaths injured at the paranode or center of the internode, in the first hour after axotomy. **(D)** Summed microglial engagement in the first hour after axotomy. Data points are colored by animal. **(E)** Frequency of myelin sheath clearance when axotomy targets myelin sheath.

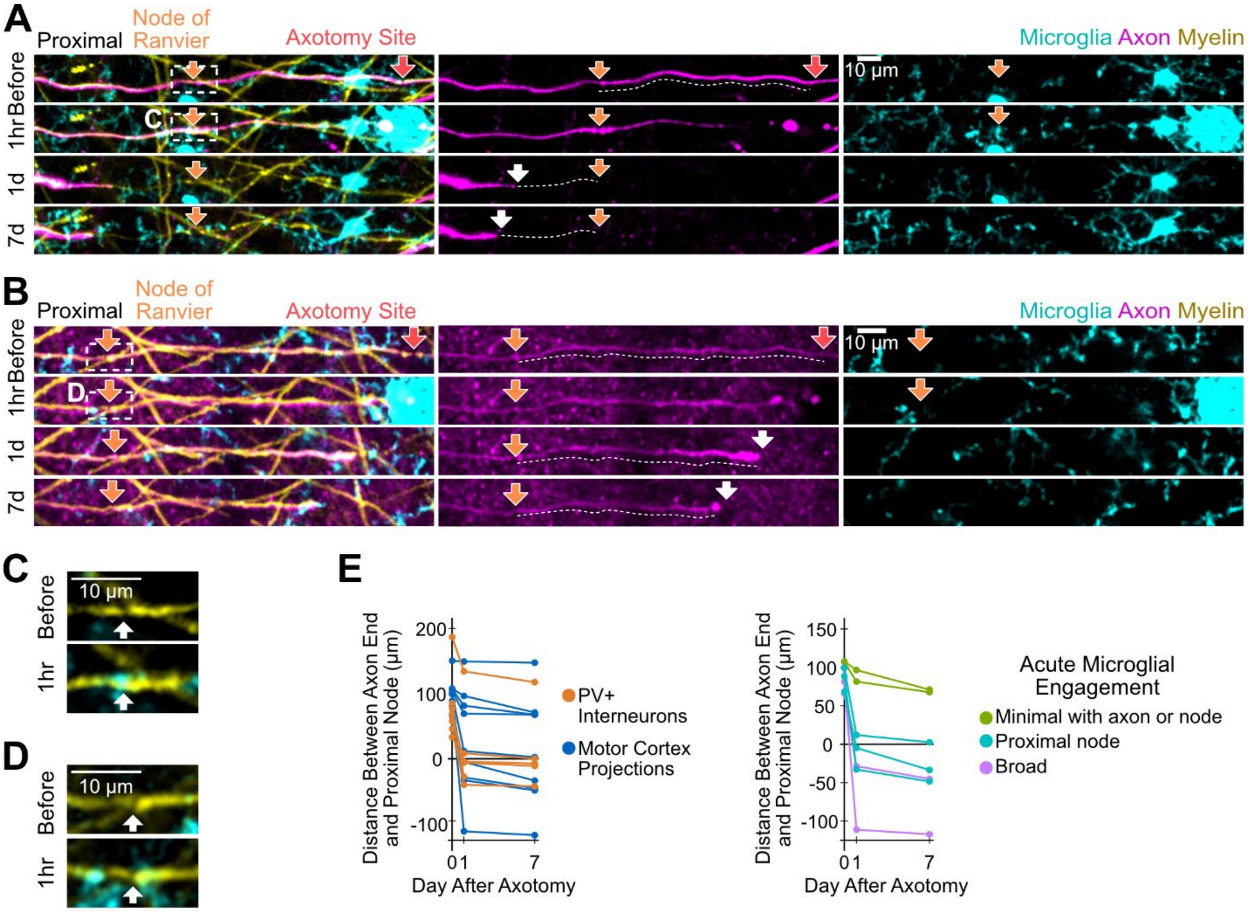

Figure S8: Retraction of myelinated axons. (A) Image series shows acute microglial engagement at the proximal node of Ranvier (orange arrow) and subsequent proximal axon retraction. The dashed line indicates the measurement for (E); the distance between the axon end and the proximal node of Ranvier. **(B)** Similar to (A) for an axon that lacks acute microglial engagement at the proximal node of Ranvier. The axon shown here is also shown in Fig. 5B, left. **(C)** Box from (A) showing node of Ranvier (white arrows) with microglial engagement 1 hour after axotomy. **(D)** Box from (B) showing nodes of Ranvier (white arrows) without microglial engagement. **(E)** On the left, proximal axon retraction relative to the proximal node of Ranvier for PV+ axons and axons projecting from the motor cortex. This same measurement is shown on the right, for motor cortex projections colored by pattern of acute microglial engagement, when it could be measured.

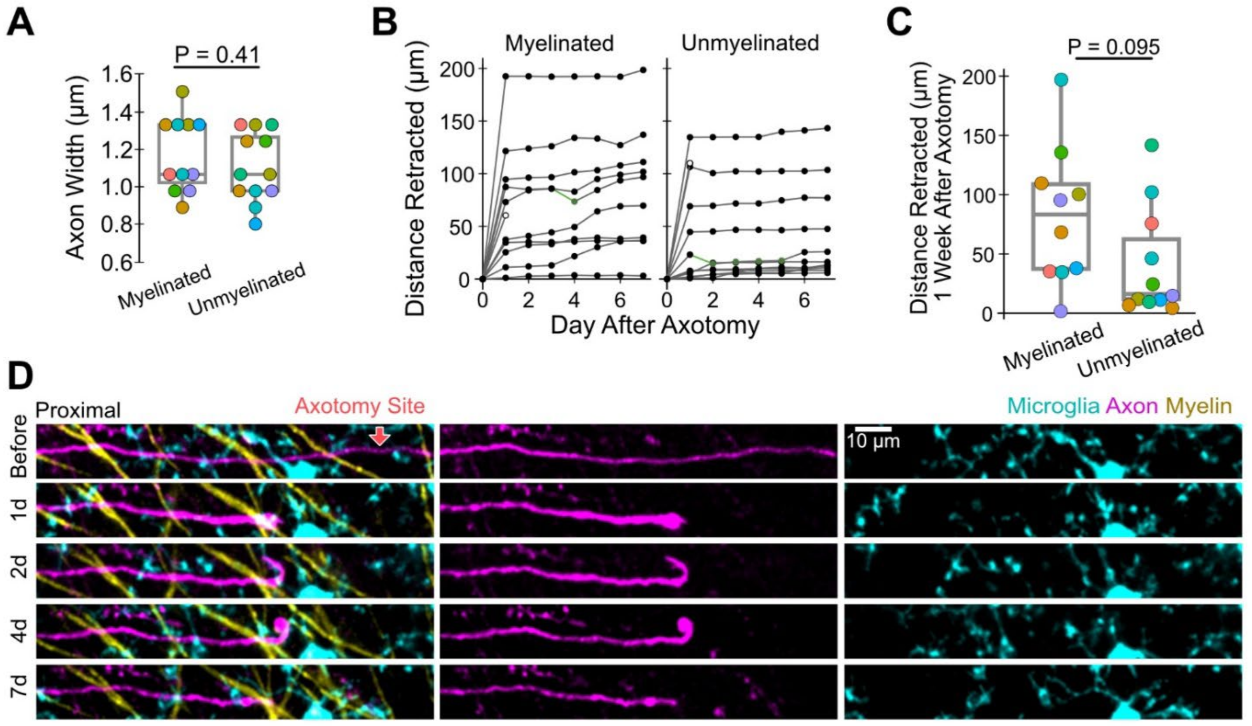

Figure S9: Myelination Status is unimportant for proximal axon retraction. (A) Axon width as determined with FWHM for axons selected for axotomy (n = 11 myelinated and 12 unmyelinated axons from 8 mice, significance tested with Welch's t-test). Data points are colored by animal. **(B)** Distance retracted from the axotomy site for the proximal ends of each axon. Green points and lines indicate regenerative sprouting. Open points indicate axons that retract outside the imaging field of view (n = 11 myelinated and 12 unmyelinated axons from 8 mice). **(C)** Distance retracted by proximal axons did not significantly vary between myelinated and unmyelinated axons (n = 11 myelinated and 12 unmyelinated axons from 8 mice, significance tested with Welch's t-test). Data points are colored by animal. **(D)** Image series shows the proximal end of a targeted unmyelinated axon with a transient regeneration attempt. Detailed statistical results in Data S1.

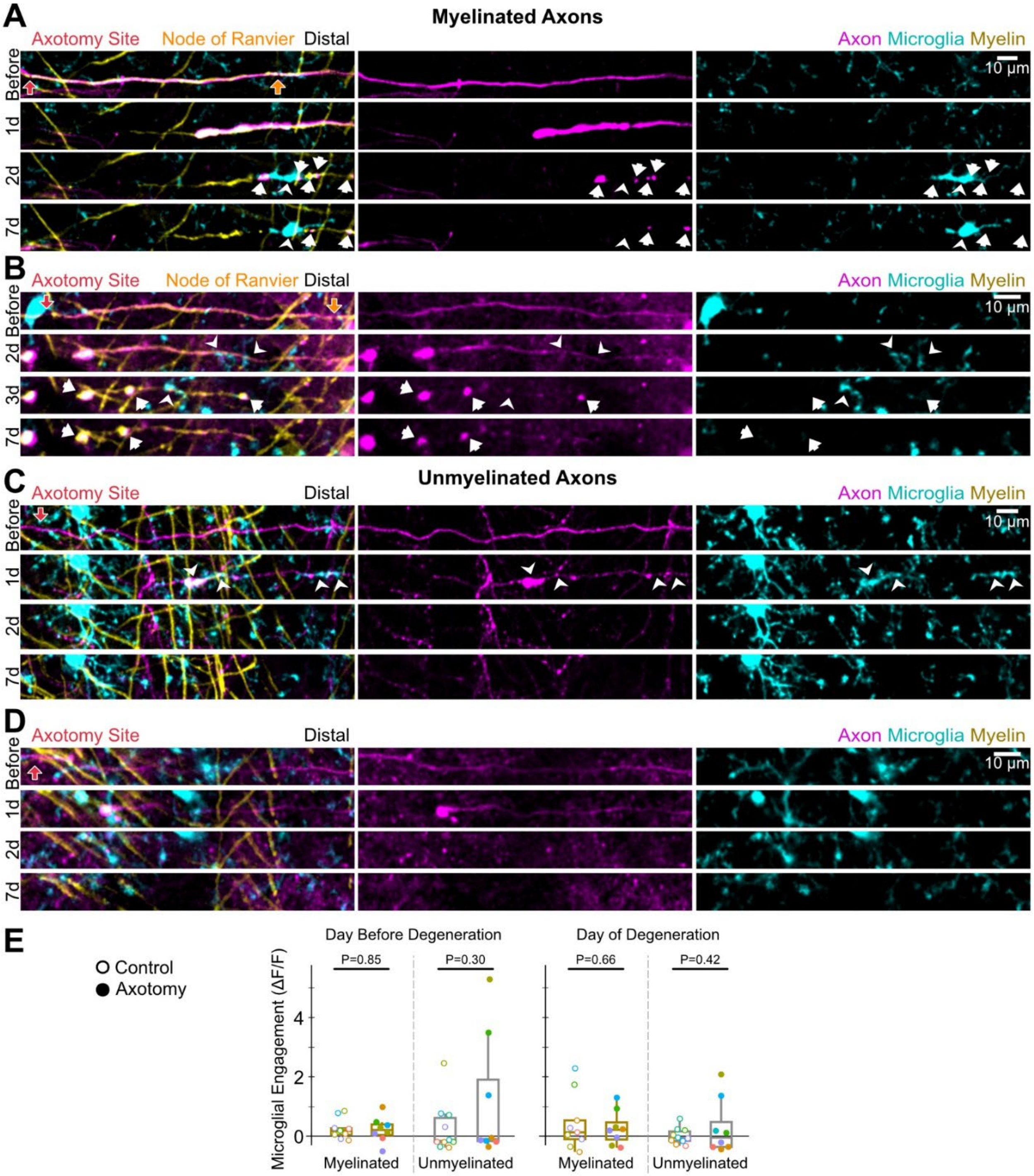

Figure S10: Microglia rarely engage with axons during Wallerian degeneration. (A) and **(B)** Image series shows acute retraction and later degeneration of axons distal to the axotomy site, for myelinated axons. White arrowheads point to microglial engagement with axons, myelin, or in a region that was previously a node of Ranvier (A). White arrows point to axon debris remaining after axon degeneration. **(C)** and **(D)** Similar to (A) and (B) for unmyelinated axons. **(E)** Figure 6G with data points colored by animal.

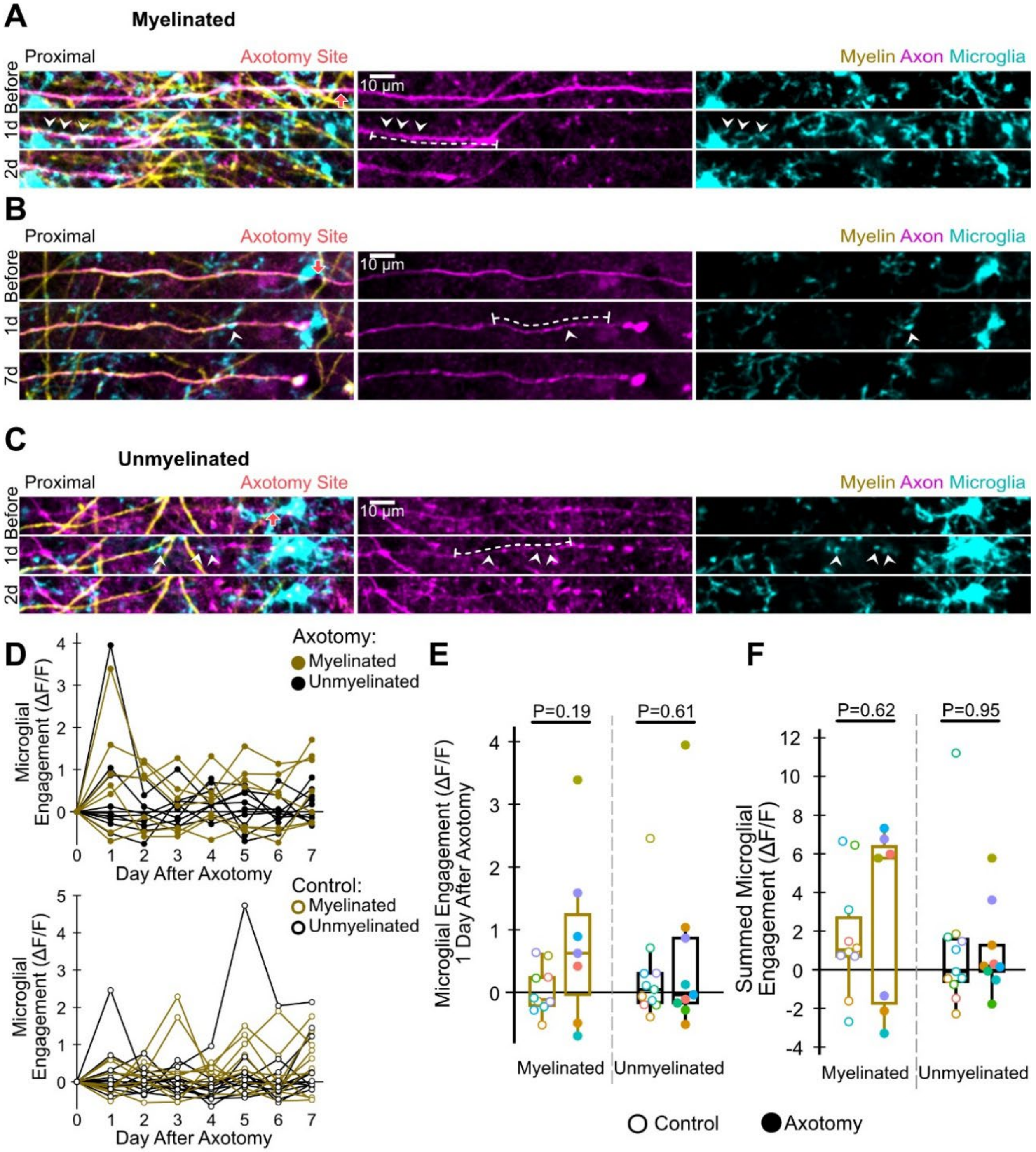

Figure S11: Microglia rarely engage with the proximal stumps of axotomized axons. (A) and **(B)** Image series shows microglial engagement with proximal stumps of myelinated axons after axotomy. The dashed line indicates 50 μm ROIs aligned with the axon stump at least 15 μm away from the axotomy site, used for quantification of microglial engagement. White arrowheads point to regions of transient microglia engagement. **(C)** Similar to (A) and (B) for an unmyelinated axon. **(D)** Graph on the top shows traces of microglial engagement with proximal axon stumps in the week following axotomy. Graph on the bottom shows traces of microglial engagement with

uninjured control axons. **(E)** Microglial engagement with proximal axon stumps 1 day after axotomy (significance tested with Welch's t-test). Data points are colored by animal. **(F)** Summation of daily microglial engagement with proximal axon stumps in the week after axotomy (significance tested with Welch's t-test). Data points are colored by animal. For (D), (E), and (F), n = 7 axotomized myelinated, 10 control myelinated, 9 axotomized unmyelinated, and 11 control unmyelinated axons from 8 mice. Detailed statistical results in Data S1.

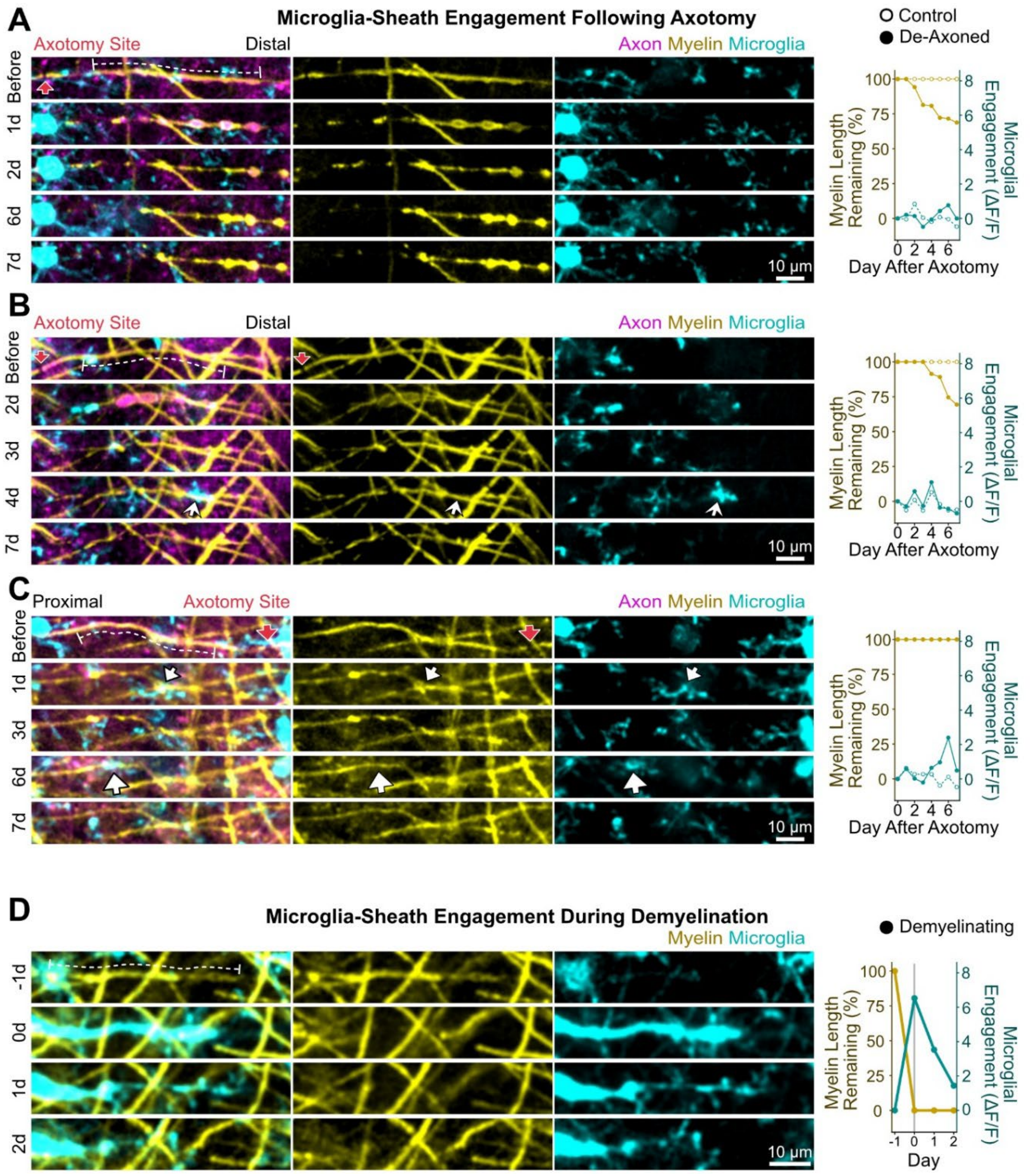

Figure S12: Microglia do not respond to de-axoned myelin. (A), **(B)**, and **(C)** Image series show microglial engagement with de-axoned myelin sheaths. Microglia engagement was analyzed in 50 μm ROIs starting 15 μm away from the axotomy site (white dashed lines). White arrows point to regions of transient microglial engagement, not associated with myelin clearance. On the right, myelin length remaining is overlaid with microglial engagement for de-axoned myelin sheaths and control myelin sheaths in the same imaging position. **(D)** Similar to (A), (B), and (C), showing microglia-mediated clearance myelin sheath during demyelination.
